# Echoes of the spoken past: how auditory cortex hears context during speech perception

**DOI:** 10.1098/rstb.2013.0297

**Published:** 2014-09-19

**Authors:** Jeremy I. Skipper

**Affiliations:** Department of Cognitive, Perceptual and Brain Sciences, Institute for Multimodal Communication, University College London, London, WC1H 0AP, UK

**Keywords:** auditory, context, brain, language, multimodal, predictive

## Abstract

What do we hear when someone speaks and what does auditory cortex (AC) do with that sound? Given how meaningful speech is, it might be hypothesized that AC is most active when other people talk so that their productions get decoded. Here, neuroimaging meta-analyses show the opposite: AC is least active and sometimes deactivated when participants listened to meaningful speech compared to less meaningful sounds. Results are explained by an active hypothesis-and-test mechanism where speech production (SP) regions are neurally re-used to predict auditory objects associated with available context. By this model, more AC activity for less meaningful sounds occurs because predictions are less successful from context, requiring further hypotheses be tested. This also explains the large overlap of AC co-activity for less meaningful sounds with meta-analyses of SP. An experiment showed a similar pattern of results for non-verbal context. Specifically, words produced less activity in AC and SP regions when preceded by co-speech gestures that visually described those words compared to those words without gestures. Results collectively suggest that what we ‘hear’ during real-world speech perception may come more from the brain than our ears and that the function of AC is to confirm or deny internal predictions about the identity of sounds.

## 1. Introduction

…whensoever any sound agitates the Brain, there flow immediately spirits towards the Muscles of the Larynx, which duly dispose them to form a sound altogether like that, which was just now striking the Brain.

[1, pg. 9]

The hearing ear is always found close to the speaking tongue.

[2, pg. 53]

### (a) What do we hear and what does auditory cortex do with speech?

Language use is paramount to our social, emotional and even physical well-being [3]. It underpins most of our cognitive processes, including learning, reasoning, problem solving and decision-making. Understanding the organization of language and the brain, therefore, is critical for understanding the mechanisms that support these processes and for fixing them when disorders occur. A reasonable place to start, at least for spoken language, would seem to be with audition and the role of auditory cortex (AC) in hearing speech. What do we hear when we listen to someone speak? What does AC do with that speech? These might seem like antiquated questions with obvious answers like ‘we hear sounds’ and ‘AC does something relatively low-level, like extract acoustic features’. The feeling that we hear speech sounds coming from our ears seems to follow unambiguously from our phenomenological experience. That AC extracts acoustic features follows from the location of primary AC in the transverse temporal gyri (TTG), the first stop in the cortex in an assumed processing hierarchy starting from the outside world of moving air molecules and ending with language comprehension in ‘higher’ cortical region(s).

This characterization of the location of primary AC at the beginning of a processing hierarchy, however, is not consistent with its functional properties or connectivity. Action potentials arrive in primary AC from the medial geniculate nucleus of the thalamus roughly three to five synapses after being fired in the cochlear nerve. This means they have been through more elaborate processing than those arriving in primary visual cortex [4]. Indeed, all of the proposed features that have been claimed to be extracted by primary AC have also been shown to be extracted subcortically [5,6]. Rather, primary AC seems to do something more complex, perhaps processing ‘auditory objects’ [7,8]. Evidence includes data that primary AC neurons have many different multisensory response properties, variable temporal windows of integration that are typically longer than isolated features, respond to combinations of sounds in the same way as to a single aspect of that combination and do not represent acoustic features faithfully [4,9–12]. Finally, the feedback connectivity that exists at all levels of AC [6] is also inconsistent with primary AC simply being the start of a processing hierarchy. By some estimates, only 30% of the neurons projecting to primary AC are part of ascending inputs from subcortical structures, whereas 70% are from other cortical regions and at least 20% of those come from non-auditory regions [13–15].

There may be a relationship between the characterization of primary AC as an auditory object processor and that it has more feedback than feed-forward connectivity. That is, processing auditory objects seems to require prior knowledge (consider, for example, the inability to distinguish phonemes or words in speech spoken in a language never before heard). The feedback connections to AC may correspond to a pathway by which this knowledge can inform processing. A model is described in the next section in which those feedback connections are proposed to carry a type of knowledge: predictions about the auditory consequences of producing speech. Those predictions are said to be unconscious tests of hypotheses about what auditory objects should be heard and the information arriving in AC as evidence required to confirm or deny those hypotheses. If true, this model implies, as shall be seen, that what we hear when someone speaks is not ‘sound’ and that AC does not extract acoustic features nor is it the beginning of a processing hierarchy *per se*.

### (b) A model of the natural organization of language and the brain

#### (i) Overview

A model of the natural organization of language and the brain (henceforth, the natural organization of language and the brain (NOLB) model) is described in this section to ultimately make sense of claims about what we hear and what AC does (see [16,17] for further detail and supporting references, and see [18–21] for other neurobiological models of language incorporating prediction). This model was originally proposed to account for the ‘lack of invariance’ problem, referring to the lack of one-to-one mapping between the acoustic patterns of speech and what we hear [17]. Variance includes many-to-one mappings in which different acoustic patterns give rise to the perception of the same sound and one-to-many mappings where one acoustic pattern gives rise to different percepts. In the history of the study of speech perception, no acoustic features have been found that invariably specify any given acoustic pattern as a particular hypothetical speech category (like a phoneme) [22]. We suggested that this non-deterministic mapping problem can be solved by considering language from a natural, ecological or real-world perspective and positing that speech perception is an active process that uses information uniquely available in those settings to achieve perceptual constancy.

In particular, during natural language use there is an abundance of contextual information available to listeners that is not present in the experimental preparations typically used to study speech. Context can be defined as any information surrounding (though likely mostly preceding) a focal sound pattern that could play a role in determining its meaning. Such information can serve as context along a time-varying continuum, from external to internal to the listener. External context is multimodal and encompasses the physical situation of language use, for example, audible verbal and observable non-verbal information like speech-associated mouth movements, co-speech gestures and body posture. For external context to be used in determining the meaning of sound patterns, it must be associated with internal knowledge established through learned experience. Such knowledge can then serve as context itself as when, for example, ‘hear’, ‘happy’ and ‘cat’ ultimately serve as internal context for ‘purring’ in ‘Can you hear the happy cat…’.

We proposed that listeners can use context in an active and predictive way because of the timing of and learned probabilistic associations between various types of context and accompanying auditory objects. For example, observed speech-associated mouth movements and co-speech gestures occur before the sounds that accompany those movements. Because of learned associations between the observed movements and the auditory objects that follow, mouth movements can activate phonemes whereas gestures can activate the words associated with those movements in advance of their occurrence. These pre-activated associated auditory objects then serve as hypotheses in a process of ‘unconscious inference’ [23] where an hypothesis is ‘tested’ by comparing it to the acoustic patterns arriving soon after from the ears. A predicted object can be confirmed or denied even though the acoustic patterns themselves lack invariance. That is, by analogy with the scientific method, hypotheses put constraints on how data are interpreted despite that information being inevitably impoverished (e.g. incomplete, noisy or variant).

#### (ii) Neurobiological specification

The NOLB model has a set of organizing neurobiological principles (see [16] for more details). First, the NOLB comprises many distributed and simultaneously active networks. Each network is composed of a set of regions or nodes operating in synchrony and organized around available context and *not* traditional linguistic units of analysis like phonemes, syllables or words *per se* (though those could be context). Individual networks are dynamic and self-organizing because the context available during natural language use is ever changing. Each network can be partially or fully ‘reinstated’ by activation of any one of its nodes through spreading activation. For example, an observed iconic ‘flapping’ co-speech gesture might comprise a network (formed through Hebbian-like associative processes [24]) that includes distributed nodes involved in the representation of observed hand and arm movements (e.g. parietal and premotor cortex for ‘mirroring’ those movements), semantic features (e.g. inferior temporal visual object representations of birds) and auditory objects associated with those features (e.g. AC representations of the word ‘bird’). Which of these nodes is reinstated is said to be a function of the informativeness of the context, observers' experience with such context (in this case with gestures and birds) and what other context is available and the networks associated with those other forms of context. The latter can occur because networks share nodes allowing all active networks to cooperate (and compete). To illustrate, imagine someone saying ‘Is it an airplane or a…’ where ‘bird’ nodes are minimally activated in association with ‘airplane’. If that person also produces the ‘flapping’ gesture, ‘bird’ nodes will become even more active through shared nodes between networks. That is, these networks cooperated to weight the ‘bird’ nodes more strongly, increasing the synchrony of that network.

**Figure 1.**
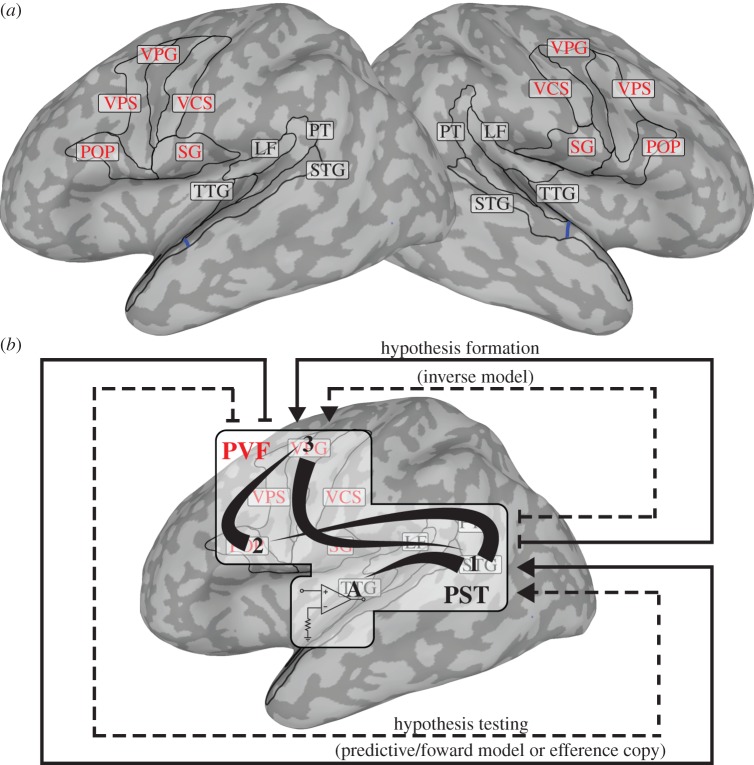
Regions supporting and visualization of the active hypothesis-and-test mechanism associated with the NOLB model (see §1*b* for details). (*a*) Posterior superior temporal (PST) regions (black letters) include the transverse temporal gyrus/sulcus (TTG), posterior aspect of the lateral fissure (LF), planum temporale (PT) and superior temporal gyrus (STG). Though the entire STG is drawn for reference, PST regions include only cortex posterior to the blue line drawn at the anterior aspect of the TTG. Posterior ventral frontal (PVF) regions (red letters) include the pars opercularis (POP) of the inferior frontal gyrus, ventral aspects of the precentral sulcus (VPS), precentral gyrus (VPG) and central sulcus (VCS) and the subcentral gyrus and sulcus (SG). (*b*) Visualization of hypothesis-and-test processing steps associated with these regions. Hypotheses are formed and tested through bidirectional network interactions between PST and PVF regions. Specifically, context is used to generate hypotheses about associated auditory objects in PST regions (1), an hypothesis is specified as a motor goal and mapped onto motor plans to produce (speak) that goal in PVF regions (1 → 2 → 3), the auditory object associated with those plans is activated through feedback (3 → 1) and compared with acoustic patterns arriving in the TTG (1 → A, represented by the ‘comparator’). A difference results in an error signal, and 1–3 and A are repeated until the error signal is suppressed. A strong prediction might result in a small error signal and only one cycle through the hypothesis formation and testing network (dashed lines). A weaker prediction might require multiple hypothesis-and-test cycles and more metabolic expenditure (dashed and solid lines).

The focus of this manuscript is on the principles associated with the NOLB model that pick up where the above description leaves off. That is, each network is said to implement the active mechanism described in general terms in the prior section with the resulting behavioural consequence being perceptual constancy and, as we shall see, the associated neurobiological consequences being an increase in processing speed and metabolic savings. Specifically, this active mechanism is implemented through the ‘neural reuse’ [25] of brain regions supporting speech production (SP). This is because SP regions can already (i) translate between desired auditory objects and the steps needed to produce associated sounds and (ii) monitor vocalizations by a feedback mechanism in which the sensory consequences of the produced movements are compared with auditory input. Such monitoring is likely necessary for vocal learning and rapid vocal adjustment to real-time perturbations [26]. When auditory objects associated with different contexts are specified as a SP plan, they can re-use this monitoring mechanism to provide a constraint on the interpretation of the (non-deterministic) acoustic patterns arriving in AC. The processing steps and associated AC and SP regions supporting this active process are described next and visualized in figure 1. These steps are intended to be a simplification of the more continuous network-based and less stage like processes likely implemented in the brain. As a simplification, some ‘core’ regions supporting SP, like the anterior insula, supplementary motor area (SMA), basal ganglia and cerebellum [27,28], are not explicitly considered until the discussion (§4*b*(i))

*Hypothesis formation* (figure 1*b* 1 → 3)*.* An auditory object serves as an hypothesis (picking up on the prior example, ‘bird’ now has the highest level of synchrony and serves as the hypothesis). These auditory objects likely correspond to activity in posterior superior temporal (PST) regions defined in figure 1*a*. The hypothesized auditory object is mapped onto the motor goal that would be associated with producing (speaking) that object (perhaps mediated by inferior parietal cortices). This is sometimes called an ‘inverse model’ and may involve arcuate fasciculus fibre track connections to the pars opercularis and nearby regions. This motor goal is then mapped to a specific motor ‘plan’ that could be used to reach that goal. This likely involves premotor and primary motor cortices given the somewhat somatotopic representation of the articulators in these regions [29,30]. These SP-related processes collectively correspond to activity in posterior ventral frontal (PVF) regions as defined in figure 1*a*.

*Hypothesis testing* (figure 1*b* 3 → A)*.* Next, the prediction of the auditory object associated with executing those motor plans is engaged through feedback. This shapes processing and increases sensitivity to acoustic patterns associated with the predicted auditory object [31,32]. This is sometimes called an ‘efference copy’ or a predictive or ‘forward model’ and originates in PVF regions and may follow the same fibre bundle that the hypothesis took to reach frontal cortex back to PST regions. Then the predicted auditory object in PST regions (/b/ in the ‘bird’ example) is subtracted from the actual AC input pattern (‘b’) resulting in an error signal. If the error signal is greater than zero, it implies that something in the input still needs to be explained and a revised or new hypothesis is tested. Forward and backward propagation continues between PST and PVF regions until the error signal is suppressed.

*Metabolic savings.* The achievement of perceptual constancy through this active mechanism has the further neurobiological consequences of altering processing times and use of metabolic resources. Specifically, the stronger the hypothesis and associated prediction, the earlier that prediction can be confirmed (i.e. the earlier the prediction error is around zero). This has the functional effect of speeding up processing and perception given that, e.g. words can be identified earlier than by a purely feed-forward mechanism. In addition, the stronger the hypothesis and prediction and earlier that prediction can be confirmed, the more free cycles become available (i.e. there is no further forward and backward propagation in the SP network). These free cycles amount to conversed metabolic resources that can be used for other purposes.

#### (iii) Evidence from speech-associated mouth movements

Much of the evidence for the active processing mechanism associated with the NOLB model originally came from studies of speech-associated mouth movements. These are a visible form of context that listeners use to improve speech perception, equivalent to removing up to 20 dB of noise from the auditory signal [33]. Mouth movements occur 100 and 300 ms before the onset of the auditory information they accompany [34] and, therefore, can be used to form hypotheses to predict associated sounds. Using functional magnetic resonance imaging (fMRI), we showed that observed speech-associated mouth movements activate cortical regions supporting SP, the same regions that were active when participants produced the observed mouth movements [35,36]. We also showed that activity in PVF overlapping with SP regions corresponded to an hypothesis about the identity of the mouth movements and not a veridical representation of those movements [36,37]. Feedback from this motor hypothesis to PST regions supporting audition ultimately determined what listeners heard [36]. We argued, therefore, that the predictive ability of the motor system is used during audiovisual speech perception to lend weight to a particular interpretation of forthcoming acoustic patterns [17,35,36]. This predictive account is supported by other fMRI [38,39] and transcranial magnetic stimulation [40] studies and methods with better temporal resolution [41]. Furthermore, it has also been shown that speech-associated mouth movements speed up and reduce the amplitude of electrical brain signals elicited by audiovisual speech [42,43], suggesting that prediction leads to metabolic savings.

### (c) Summary and natural organization of language and the brain model predictions

To summarize, AC is characterized here as an auditory object processor with an abundance of feedback connectivity. It is proposed that these connections carry predictions about the nature of acoustic patterns arriving in AC. In particular, we proposed that the host of multimodal forms of context that accompany natural language are used by the brain to generate hypotheses about what auditory objects should be heard. These hypotheses are tested in a predictive way by neurally reusing an existing feedback circuit between PVF and PST regions. Predictions shape and constrain the processing done in PST regions so that perceptual constancy can be achieved.

Three *predictions* deriving from this, the NOLB model, pertaining to what we hear and what AC does with speech were tested here (note that, for clarity, ‘*predictions*’ and ‘*hypotheses*’ are henceforth italicized when referring to actual scientific tests). These *predictions* were tested with two sets of neuroimaging meta-analyses (§2) and a neuroimaging experiment (§3). They are (i) PST regions will be *less* active following meaningful linguistic compared with less meaningful stimuli and tasks (§2*b*), (ii) PST regions will be more active and form a stronger network with PVF regions also involved in SP following less meaningful stimuli and tasks (§2*c*) and (iii) a similar pattern of less PST and PVF region activity will occur for words following meaningful non-verbal context compared with the absence of that context (§3). The rationale behind these *predictions* are explained in detail in each corresponding section below.

A confirmation of the *predictions* would lead to quite different answers to the questions of what we hear and what AC does with speech that were asked at the start of the introduction. It would suggest that we do not hear ‘sounds’ but, rather, a mixture of externally generated sound patterns and internally generated auditory objects that derive from our own past experience of producing speech. That mix would vary as a function of the strength of the hypotheses that the brain formulates. When hypotheses and associated predictions are strong, what we hear would be almost entirely internally generated. This further suggests that what AC does, rather than extract acoustic features *per se*, is to confirm or deny hypotheses about what acoustic patterns will be heard and would often be, therefore, the end rather than the beginning of a processing hierarchy for actively using prior knowledge to constrain AC processing.

## 2. Meta-analyses

The first set of neuroimaging meta-analyses (§2*b*) test *prediction* (i) that meaningful linguistic stimuli and tasks will elicit less PST activity than less meaningful stimuli and tasks (e.g. words < matched pseudo-words). This is because more meaningful linguistic content is better learned and, because of this, contains more preceding information, e.g. phonemes, syllables or letters within words and other words in sentences, which can serve as context for forthcoming content. For example, when participants hear ‘trom…’, ‘bone’ would be more predictable than ‘tron’ in the non-word equivalent ‘bometron’. Thus, predictions about forthcoming acoustic patterns will be more accurate from meaningful linguistic content, resulting in less processing demands and a conservation of metabolic resources in PST regions.

The second set of meta-analyses (§2*c*) test the related *prediction* (ii) that PST will be tightly coupled with PVF regions also involved in SP, particularly for less compared with more meaningful linguistic stimuli and tasks. This is because less meaningful content has less information that can serve as context and, therefore, predictions about forthcoming acoustic patterns will be less accurate. The result is a larger error signal and more hypotheses formation and testing until the error signal is suppressed. This should involve greater activity in PST regions and a co-active network that includes PVF regions also involved in SP because of the proposed reciprocal role of these regions in the hypothesis-and-test mechanism. Conversely, meaningful linguistic content should result in less PST and PVF–SP region activity because of the availability and use of relatively more meaningful context, leading to smaller error signals and, therefore, less forward and backward propagation between PST and PVF regions.

### (a) General methods

*Predictions* (i) and (ii) were tested by searching the BrainMap database using sets of metadata criteria common to all (e.g. participant information) and specific to each hypothesis (e.g. stimulus modalities, experimental paradigms, cognitive domains and brain regions). The database queries returned *x*/*y*/*z* stereotaxic coordinate space ‘locations’, i.e. centres of mass or peaks of functional brain activity reported in neuroimaging papers corresponding to each set of metadata criteria (http://brainmap.org/) [44–46]. At the time of analyses (ending February, 2014), the database contained 2390 papers, 46 366 participants and 91 039 locations. Common metadata search criteria were used to exclude deactivations (i.e. activity below baseline) and participants who were diagnosed with a disease or disorder, left handed and younger than age 18. Electronic supplementary material, §2*a*–*d* contains the exact search criteria used for the common and more specific searches associated with each meta-analysis. Electronic supplementary material, table S1 contains an exact breakdown of the search result by the number of papers, participants, experiments, conditions and locations.

Locations originally published in the Talairach coordinate space were converted to Montreal Neurological Institute (MNI) space [47,48]. Then activation likelihood estimation (ALE) meta-analyses were done by modelling each MNI location as a three-dimensional probability distribution and quantitatively assessing their convergence across experiments. Significance was assessed by permutation analysis of above-chance clustering between experiments [28,49–51]. Contrasts or conjunctions between ALE meta-analyses were done using 10 000 permutations to derive *p*-values [50]. All resulting ALE maps were false discovery rate (FDR) corrected for multiple comparisons to *p* < 0.05 and further protected by using a minimum cluster size of 160 mm^3^ (20 voxels). These maps are displayed on inflated surface representations of an MNI-aligned template brain so that activity in sulci and on gyri can be easily visualized. PST and PVF regions proposed to support the active mechanism associated with the NOLB model are drawn on this brain (see figure 1 and the electronic supplementary material, §1 for details pertaining to surface inflation and parcellation into regions).

### (b) Auditory cortex

‘AC’ meta-analyses test *prediction* (i) that PST activity will decrease as stimuli and tasks become more meaningful. This is because more meaningful stimuli and tasks have more predictable content because they are more well learned. Meaningfulness is defined as relative to the comparisons being made. For example, ‘phonology’ is more meaningful when compared with ‘tones’ but less meaningful when compared with ‘semantics’.

#### (i) Analysis 1: transverse temporal gyrus

It is *predicted* that, when activity is reported directly in the TTG, it will be reported more in papers *not* in the more meaningful behavioural domain of language compared with some other (less meaningful) domain.

*Methods.* BrainMap was searched using the common search criteria and the further criterion that reported statistical contrasts (‘experiments’) have a location in the TTG. Each experiment with a TTG location was classified as to whether associated stimuli were audible or not and experiments were labelled with the behavioural domain of language or not. A follow-up analysis was done by classifying stimuli as containing meaningful semantic linguistic information or not (e.g. words versus pseudo-words). This is an alternative to the behavioural domain of language classification to account for the possibility that stimuli and tasks used in language studies may not generally be semantically meaningful. Count data were analysed with *χ*^2^ statistical tests.

*Results.* The TTG search returned 164 experiments. Of those, 24 (14.6%) used auditory stimuli and were classified with the behavioural domain of language, 55 (33.5%) auditory and non-language, 24 (14.6%) non-auditory and language and 61 (37.2%) non-auditory and non-language. Though the TTG is similarly activated by auditory and non-auditory stimuli (48% versus 52%), it is significantly more activated in studies labelled with a behavioural domain other than language compared with those labelled as language (71% versus 29%, *χ*^2^ = 28.20, d.f. = 1, *p*-value < 0.0000001). The follow-up analysis showed similar results with 108 (66%) of contrasts not using and 56 (34%) using meaningful linguistic stimuli (*χ*^2^ = 16.49, d.f. = 1, *p*-value < 0.00005).

#### (ii) Analysis 2: passive listening

Analysis 1 is limited because the TTG may be active despite the fact that locations are not explicitly reported there. For example, a reported peak location might be near but not in the TTG though the (unreported) centre of mass is in it. Thus, when the peak is given a three-dimensional probability distribution as part of the ALE analysis, the TTG activity would manifest if it converges across experiments. In this section, therefore, the location constraint is removed. It is *predicted* that passive listening to words will engage the PST regions (including the TTG) less than less meaningful non-words. Passive listening was chosen because it is a natural task that uses a relatively homogeneous set of stimuli that should engage AC and does not explicitly involve differential task demands. It was also *predicted* that PST regions form an interconnected network (perhaps including PVF regions) during passive listening and that this network would be less correlated with words than less meaningful stimuli.

*Methods.* The database was searched for locations contributed by the common criteria and studies in which participants passively listened to words or auditory non-words (either clicks, noise, pseudo-words, reversed speech, syllables or tones). Music and environmental sounds were excluded from the latter search because they explicitly contain meaningful content. ALE meta-analyses were done with the passive listening to word and non-word location results and then directly statistically contrasted.

A network analysis was done by re-examining a previous analysis conducted by Laird *et al*. [52] of *all* of the neuroimaging data in the BrainMap database (data are available for download at http://brainmap.org/). Specifically, Laird *et al*. [52] used independent components analysis to blindly decompose 8637 experiment images from 31 724 participants and 69 481 activation locations into spatially co-occurring intrinsic connectivity network maps. The functional specializations of each of the resulting maps were quantified by examining the per-experiment contributions of BrainMap metadata to each component. Here, networks were located that had a moderate or stronger correlation (i.e. *r* > 0.30) with the paradigm class of passive listening. The resulting map(s) were examined to determine where activity was located and what ‘stimulus type’ metadata correlated most with those maps.

**Figure 2.**
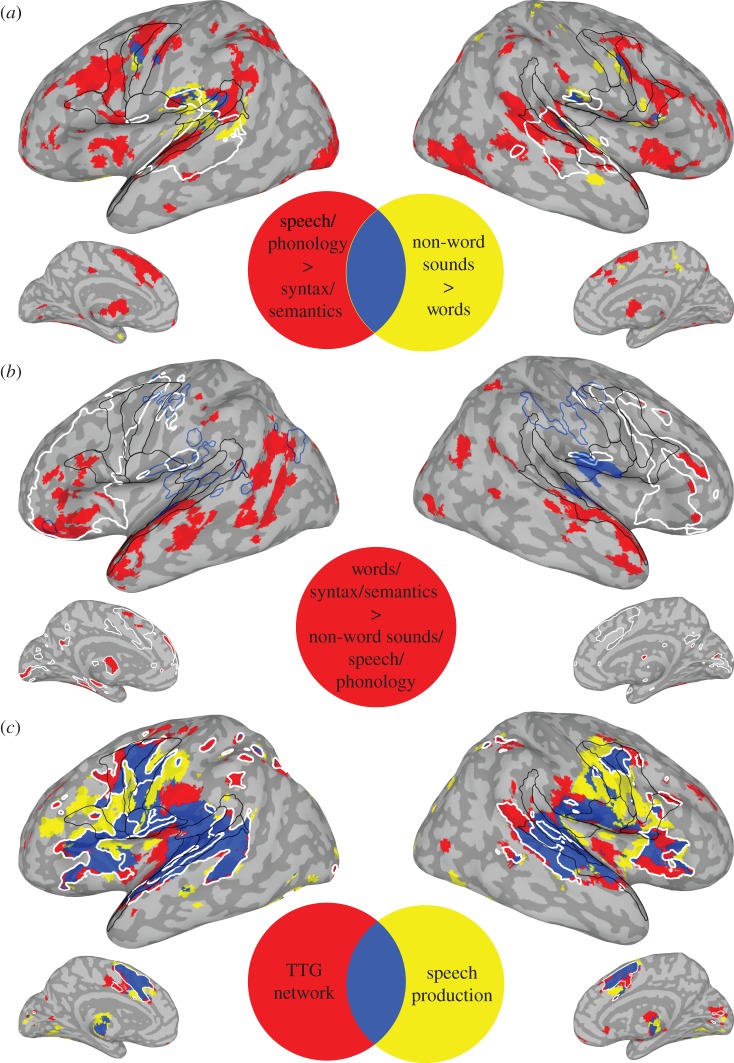
Neuroimaging meta-analyses results. (*a*) Less meaningful > meaningful stimuli and tasks: passive listening to non-words > words (yellow), experiments in the behavioural domains of language and speech or phonology > syntax or semantics (red) and their overlap (blue). White outline is the intrinsic connectivity network correlated with passive listening and less meaningful stimuli, thresholded at *z* ≥ 10 to show the PST distribution of activity. (*b*) Meaningful > less meaningful stimuli and tasks: the converse of (*a*), i.e. passive listening to words > non-words and syntax or semantics > speech or phonology (red). White outline is frontal activity for passive listening to words and experiments in the behavioural domains of syntax and semantics prior to contrast analyses. Blue outline is deactivation for auditory presentation of words and experiments in the behavioural domain of syntax and semantics. Filled in blue outline is significantly greater for the latter compared with auditory presentation of non-words, speech and phonology. (*c*) Transverse temporal gyrus (TTG) co-activation network (red), SP (yellow) and their overlap (blue). White outline is covert SP. Black outlines are PST and ventral frontal (PVF) regions as defined in figure 1*a*. All *p*'s ≤ 0.05 FDR corrected for multiple comparisons with a cluster size of 160 mm^3^ (20 voxels).

*Results.* The contrast of passive listening to words and non-words shows that words result in less and non-words in more activity in PST and PVF regions (figure 2*a*, yellow and blue, and tables 1*a* and 2*a*).

**Table 1.** Meta-analyses of activity by region. (Volume of activation in grey matter per region in mm^3^. Grey and red outlines correspond the PST and PVF regions in figure 1*a*, where region abbreviations are defined. LH and RH are left and right hemispheres, respectively. Online version in colour.)

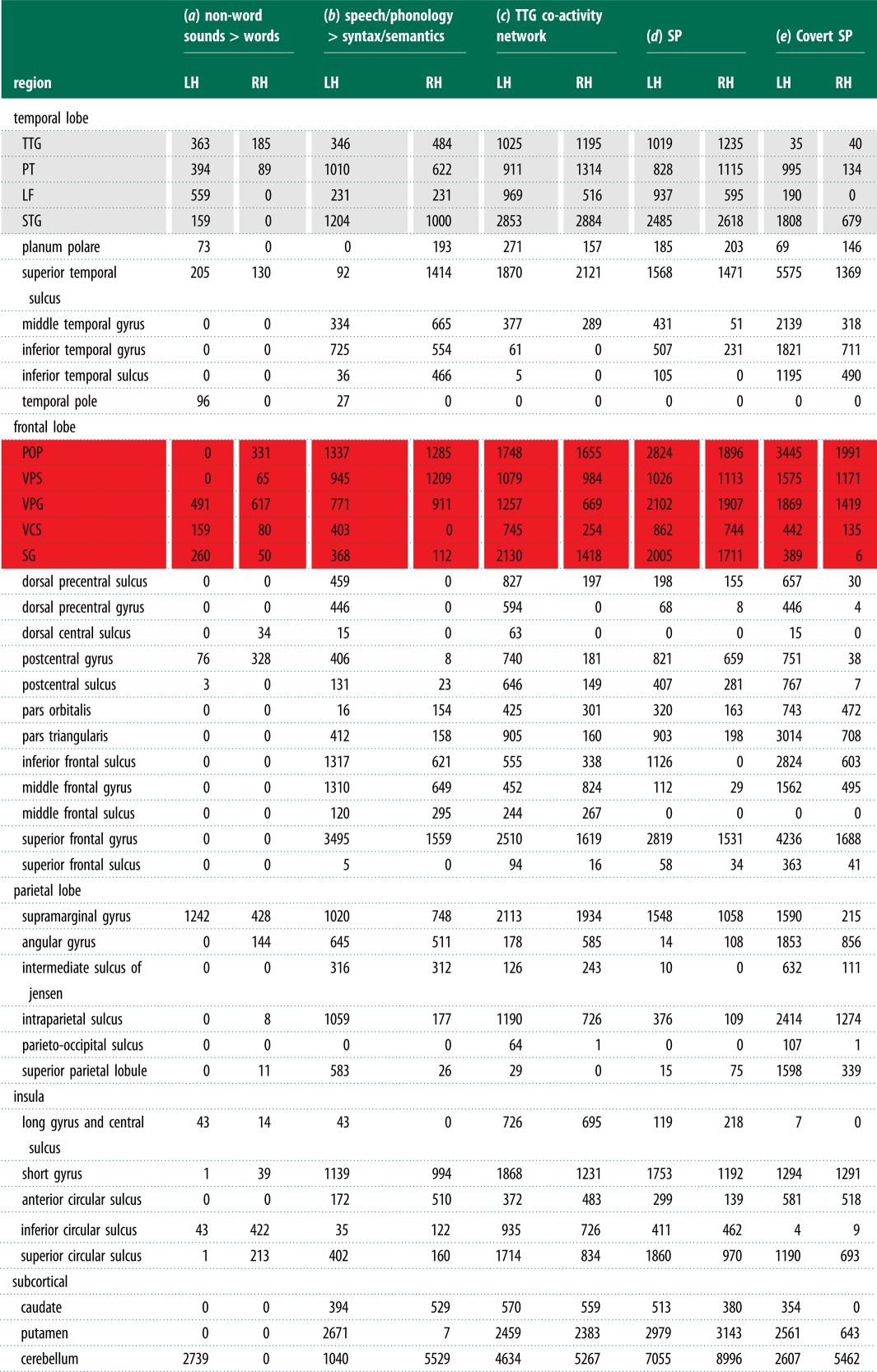

**Table 2.** Meta-analyses of activity by cluster. (Ten largest clusters only. Region abbreviations defined in figure 1*a*. MNI coordinates are centres of mass. First region is always the location at the centre and the other regions are part of the cluster. With the exception of the ‘anterior insula’, ‘anterior’ and ‘posterior’ are relative to the TTG (see figure 1*a* (blue line)).)

			voxels (mm^3^)	MNI		
meta-analyses	regions			*x*	*y*	*z*
(*a*) less meaningful stimuli and tasks						
	left	fusiform and cerebellum	27 608	−36	−71	−19
a combination of the following contrasts:	right	POP and VPG, VPS, VCS	22 040	47	13	17
passive listening to non-words > words	left	PT and TTG, posterior STG, LF	18 992	−56	−31	13
behavioural domain of language:	left	VPG and POP, VPS, VCS	17 880	−46	0	42
speech > syntax	left	superior frontal gyrus (SMA)	17 648	−2	16	54
speech > semantics	right	fusiform and cerebellum	16 848	39	−66	−23
phonology > syntax	right	posterior STG and TTG, PT, LF	16 736	55	−29	3
phonology > semantics	left	anterior insula, SG, putamen	14 432	−34	9	3
	left	superior parietal lobule	6672	−33	−63	50
	right	thalamus, caudate	4944	2	−2	5
						
(*b*) passive listening network						
correlation of stimuli with this network:	left	posterior STG and TTG, PT, LF, PP, middle temporal gyrus	64 584	−53	−24	2
non-vocal sounds, *r* = 0.91	right	posterior STG and TTG, PT, LF, PP, middle temporal gyrus	62 752	55	−21	0
non-verbal vocal sounds, *r* = 0.77	right	cerebellum	1360	10	−69	−34
noise, *r* = 0.52	right	VPG and VPS, VCS	1280	47	−8	47
music, *r* = 0.49	left	superior parietal lobule	1152	−14	−61	62
tones, *r* = 0.38	right	superior parietal lobule	872	14	−62	62
pseudo-words, *r* = 0.27	left	VPG and VCS	528	−48	−13	45
none, *r* = 0.26	right	paracentral lobule	496	9	−42	59
words, *r* = 0.25	right	middle frontal gyrus	280	31	−2	63
syllables, *r* = 0.25	left	middle frontal gyrus	248	−25	−7	64
false fonts, *r* = 0.22						
(*c*) more meaningful stimuli and tasks						
	left	PP and anterior STG, temporal pole, pars triangularis, pars orbitalis	25 480	−50	16	−10
a combination of the following contrasts:	left	posterior middle temporal gyrus	18 592	−46	−47	-7
passive listening to words > non-words	right	anterior middle temporal gyrus and anterior STG, temporal pole, pars orbitalis	11 328	57	6	-16
behavioural domain of language:	right	angular gyrus and posterior middle temporal gyrus	3104	56	−62	17
syntax > speech	left	anterior superior frontal gyrus	2528	−6	53	34
syntax > phonology	left	thalamus	1936	−7	−5	6
semantics > speech	right	cerebellum and fusiform	1864	34	−47	−29
semantics > phonology	right	pars triangularis	1672	51	28	17
	left	occipital pole	1248	−6	−95	−10
	left	putamen	1072	−21	8	6
(*d*) TTG co-activity network overlap with covert SP						
	left	anterior insula and POP, VPS, VPG, VCS, SG, thalamus, putamen, TTG, STG, PT, LF	51 328	−44	−2	15
	bilateral	superior frontal gyrus (SMA)	17 952	0	10	52
	left	anterior insula and POP, putamen	12 224	39	17	2
	right	posterior STG and TTG, PT, LF	7936	58	−35	5
	right	VPG and POP, VPS, VCS	6032	50	3	38
	left	superior and inferior parietal lobule	4696	−37	−50	49
	right	cerebellum	3784	29	−63	−33
	left	fusiform and cerebellum	3496	−39	−66	−27
	right	superior parietal lobule	1528	36	−55	49
	left	superior parietal lobule	1240	−22	−70	44

For the network analysis, only one intrinsic connectivity network map had a moderate or stronger correlation with passive listening (component 16 of 20, *r* = 0.84, see [52]). At *z* ≥ 3 and a cluster size of 20, most activity in this network was in two large clusters whose centres of mass were in PST regions (both within 8 mm of the TTG, figure 2*a* (white outline), table 2*b*). The next two largest clusters were in the cerebellum and PVF regions. The three stimulus types driving this network most strongly were ‘non-vocal sounds’, ‘non-verbal vocal sounds’ and ‘noise’. ‘Words’ were as correlated with the network as much as ‘none’, i.e. no stimulus presentation (see table 2*b* for correlation values). The *difference* in correlation between ‘non-vocal sounds’ and ‘words’ is *r* = 0.66. By all statistical tests comparing two overlapping correlations based on dependent groups of this size, even when estimating a high correlation between groups, this difference is highly significant (see, e.g. library(cocor) [53] in R, http://www.r-project.org/).

#### (iii) Analysis 3: speech, phonology, syntax and semantics

Perhaps the passive listening results from analyses 1 and 2 are somehow contingent on the criterion that there be no explicit task (e.g. maybe participants pay more attention to non-words). Therefore, further meta-analyses were done in which participants often performed explicit (e.g. meta-linguistic judgement) tasks that engage attention to stimuli. These task-based studies typically have a matching effort/attention control, theoretically removing this effect from the data during analysis. It was *hypothesized* that PST regions would, nonetheless, still be less activated in studies described as being in the behavioural domain of syntax or semantics compared with less meaningful speech or phonology, independent of modalities of stimulus presentation or tasks.

*Methods.* The database was searched for locations contributed by the common criteria and studies in the behavioural domain of speech, phonology, syntax or semantics, but that excluded overt oral/facial responses (because of *hypotheses* pertaining to SP regions in §2*c*). ALE meta-analyses were done for each of these behavioural domains. Then, in separate contrasts, the syntax or semantics were directly statistically contrasted with the speech or phonology meta-analyses.

*Results.* The contrast of results from experiments in the behavioural domain of syntax or semantics when compared with speech or phonology shows that syntax and semantics result in less and speech and phonology more activity in PST and PVF regions (figure 2*a* (red and blue), and tables 1*b* and 2*a*).

### (c) Speech production

The first set of meta-analyses confirm *prediction* (i) that PST regions are less robustly activated by more compared with less meaningful stimuli and tasks. In this section, two further *predictions* are tested that correspond to the proposal that the active hypothesis-and-test mechanism associated with the NOLB model re-uses SP regions and that it does so more for less meaningful stimuli and tasks. In particular, the first *hypothesis* is that the TTG will form a robust functional network with PST and PVF regions that will themselves be involved in SP. The second *hypothesis* is that less meaningful stimuli and tasks will overlap more with PVF and other regions involved in SP than more meaningful linguistic stimuli and tasks. These *hypotheses* were tested with meta-analyses of both overt and covert SP. Overt SP was used because it is fairly natural, participants cannot hear themselves well if at all during fMRI and AC is likely active independent of auditory or bone conduction feedback during production, as demonstrated in studies of covert SP [54,55]. Nonetheless, analyses were also done with covert SP to determine overlap in the clear absence of such feedback.

#### (i) Methods

To determine which regions reliably co-activated with the TTG and overlapped with SP, BrainMap was searched using the common search criteria and the three further criteria. First, the TTG search (§2*b*(i)) was repeated with the added criteria that studies involving SP were excluded. The other two search criteria were for overt SP and covert SP. The new TTG search results were used as the basis of a co-activation ALE meta-analysis, a determination of which regions reliably co-activate with the TTG [50]. The resulting TTG network was conjoined with the results of the overt SP and covert SP meta-analyses to determine the extent of overlap with each. Similarly, the combined contrasts from §2*b*(ii) to (iii) were conjoined with the overt SP and covert SP meta-analyses to determine their overlap.

#### (ii) Results

The TTG co-activation network meta-analysis highly overlapped with activation associated with both overt and covert SP meta-analyses with the largest overlap in PST and PVF regions (figure 2*c* (blue and white outline); tables 1*c*–*e*, 2*d* and 3). Similarly, less meaningful contrasts from §2*b*(ii) to (iii) showed more overlap with both overt and covert SP than more meaningful contrasts with, again, the largest overlap being in PST and PVF regions (compare activation patterns in figure 2*a*–*c* and table 3). Though more meaningful stimuli and tasks produced less or no overlap with SP regions, this pattern should not be taken to imply that these regions are inactive for more meaningful contrasts. This is evident upon examination of activity patterns in frontal cortex prior to meta-analysis contrasts (figure 2*b* (white outline)).

**Table 3.** Per cent overlap of meta-analysis with SP. (See figure 1*a* for location of the PST and PVF regions. PST and PVF overlaps were calculated using grey matter only. See table 2*a*,*c* for definitions of less and more meaningful stimuli and tasks.)

	whole brain		PST and PVF regions	
meta-analyses	overt	covert	overt	covert
TTG co-activity network	55	71	30	58
less meaningful stimuli and tasks	35	43	36	54
more meaningful stimuli and tasks	7	8	12	8

### (d) Additional analyses

#### (i) Baseline control

A follow-up analysis was conducted to confirm that results are not due to differences in the types of control stimuli and tasks used in studies contributing to meta-analyses. This was done by removing from the passive listening (§2*b*(ii)) and speech, phonology, syntax and semantics (§2*b*(iii)) searches all contrasts with control and comparison stimuli and tasks that were not a direct comparison with fixation or rest. Despite a large reduction in power compared with the original analysis, results were similar to those in figure 2*a*, showing that more meaningful stimuli and tasks result in less PST and PVF region activity. The largest cluster of activity more active for less meaningful stimuli and tasks encompassed all left hemisphere PST regions (9168 mm^3^, *x*/*y*/*z* centre of mass = −53/−37/17). The second largest cluster included activity in the right PST regions, specifically in the TTG and PT (2480 mm^3^, *x*/*y*/*z* centre of mass = 45/−20/−1). Other clusters larger than 800 mm^3^ (i.e. 100 voxels), in order of size, were in the left cerebellum, left superior parietal, left PVF regions (specifically, the VPS, VPG and CS) and right cerebellum.

#### (ii) Deactivations

Analyses have been based on relative changes in activation. Decreases in activity from baseline may be driven by a decrease in neuronal activity and corresponding energy consumption (perhaps involving reallocation of resources and/or inhibition, see [56] for discussion). If true, more meaningful stimuli and tasks might deactivate AC. To test this *hypothesis*, BrainMap was searched for the common criteria as usual, but with deactivation instead of activation. Because of the relative lack of reported deactivations in the BrainMap database (e.g. only 7% of all locations reported for the behavioural domain of language), specific search criteria combined experiments in which participants were presented words with those from the behavioural domain of language syntax and semantics. Conversely, non-word searches (either clicks, noise, pseudo-words, reversed speech, syllables or tones) were combined with speech and phonology. Furthermore, studies were restricted to the auditory modality alone because visual stimuli can lead to cross-modal decreases in activity in AC [57] and experiments without oral/facial movements because of possible suppression of AC during SP [58]. The ALE meta-analysis for more meaningful stimuli and tasks shows deactivation in PST and PVF regions bilaterally (figure 2*b* (blue outline)). Despite lacking statistical power, the direct contrast of meaningful with less meaningful meta-analyses shows larger deactivation in the right TTG for more meaningful stimuli and tasks (figure 2*b* (blue shading), 1328 mm^3^, *x*/*y*/*z* centre of mass = 48/−19/12).

### (e) Summary of meta-analysis results

Two sets of meta-analyses confirmed *predictions* (i) and (ii). Concerning *prediction* (i), AC, particularly the PST regions, is less activated by more meaningful and predictive compared with less meaningful stimuli and tasks. Specifically, analysis 1 (§2*b*(i)) showed that the TTG is less activated by language and meaningful linguistic stimuli than non-language and less meaningful stimuli. The combined results of contrasts from analysis 2 (§2*b*(ii)) and 3 (§2*b*(iii)) show that less meaningful compared to more meaningful stimuli and tasks result in more activity in primarily PST and PVF regions (figure 2*a* (all colours), compare tables 1*a*, *b* and 2*a*). The various contrasts overlapped most in the PT, VPS and VPG (figure 2*a* (blue)). Other prominent regions showing more activity for less meaningful stimuli and tasks include the inferior parietal lobules (IPLs), anterior insula, SMA, basal ganglia (i.e. the caudate and putamen) and cerebellum. Conversely, activity associated with relatively more meaningful stimuli and tasks have a different distribution of activity, engaging PST and PVF regions less and the anterior ST, middle temporal and pre-frontal regions more (figure 2*b* (red) and table 2*c*). Concerning *prediction* (ii), as already suggested by *prediction* (i) results, more activity in PST regions for less meaningful content occurred concomitantly with more activity in PVF regions also involved in SP. Specifically, there was a high amount of overlap of both the TTG co-activation network and activation associated with less meaningful stimuli and tasks with activation associated with both overt and covert SP, particularly in PST and PVF regions but also the IPL, anterior insula, SMA, basal ganglia and cerebellum (§2*c*; figure 2*c*; tables 1*c*–*e*, 2*d* and 3). Additional analysis (§2*d*) ruled out some alternative explanations concerning *prediction* (i) and (ii) results and lent further support by showing that more meaningful stimuli and tasks can also deactivate PST regions.

## 3. Experiment

‘Meaningfulness’ is only a proxy for the predictable information available in meta-analyses stimuli and tasks. Furthermore, the context associated with meta-analyses stimuli was relatively impoverished, reflecting the propensity of neuroimaging studies to use smaller and isolated unimodal (and hypothetical) linguistic units of analysis as stimuli [16]. That is, context would have primarily come from other sounds, phonemes, syllables or letters within words and, less frequently, from other words. Far more multimodal forms of context would be available for the brain to generate and test hypotheses with during natural language use. For these reasons, an experiment was conducted that directly manipulated context to provide support for the position that it is the predictive aspect of the context driving meta-analyses results. Furthermore, the experiment extends results beyond the mostly local unimodal linguistic context available in meta-analyses experiments to include an example of visual non-verbal context.

Specifically, sentence stimuli were made containing observable co-speech gestures (henceforth, gestures). Gestures are a common form of non-verbal context, involving observable movements of the hands and arms that are synchronized and co-expressive with speech [59]. Gestures benefit communication [60] and this is likely because they provide meaningful information albeit in a different (more global and visible) form from speech [61]. By one estimation, this semantic information precedes the words those gestures are co-expressive with (henceforth, lexical affiliates) by up to 250 ms with a large standard deviation of almost 500 ms [62] (see also [63]). We argued that this temporal asynchrony allows gestures to be used by the brain to predict associated lexical affiliates [17], a proposal supported by data showing that gestures can prime subsequent words and concepts [64–68].

The gestures in the experimental sentences provide preceding visual iconic (illustrating) semantic information about lexical affiliates following those gestures. They should, therefore, have the effect of making the lexical affiliate more predictable from context in the same way that preceding phonemes in more meaningful meta-analyses words, for example, would have made the ends of words more predictable when compared with less meaningful pseudo-words. Specifically, when these gestures are observable, there is more contextual information with which to formulate and test hypotheses about upcoming lexical affiliates. This results in metabolic savings at the time of that word because it has been accurately predicted, there is no error signal and no further hypotheses need to be formed and tested. Thus, like meta-analyses results, the PST and PVF regions were *hypothesized* to be less active when lexical affiliates were preceded by gestures compared with when not accompanied by those gestures. Conversely, the same words without preceding gestures should result in increased activity in PST and PVF regions also involved in SP because the hypothesis-and-test mechanism will need to be engaged at that time to use local linguistic context to process those words.

High-density electroencephalography (EEG) neuroimaging was used to test this *hypothesis* because of the precise temporal accuracy required to time lock results to the lexical affiliate within a sentence. That is, responses to a word in a sentence could likely not be deconvolved from the hemodynamic response associated with methods with good spatial resolution like fMRI without constructing unnaturally temporally jittered gestures and lexical affiliates [69]. Besides, EEG has decent spatial resolution when source localization is done with a good head model (sources are often within 10 mm of fMRI peaks, e.g. [70]).

### (a) Methods

#### (i) Participants

Forty-two (21 female, mean age = 21.13, mean Oldfield handedness score [71] = 73.94) native English speakers with normal or corrected vision and hearing participated. Participants gave written informed consent and the study was approved by the Institutional Review Board of Hamilton College.

#### (ii) Stimuli and task

**Figure 3.**
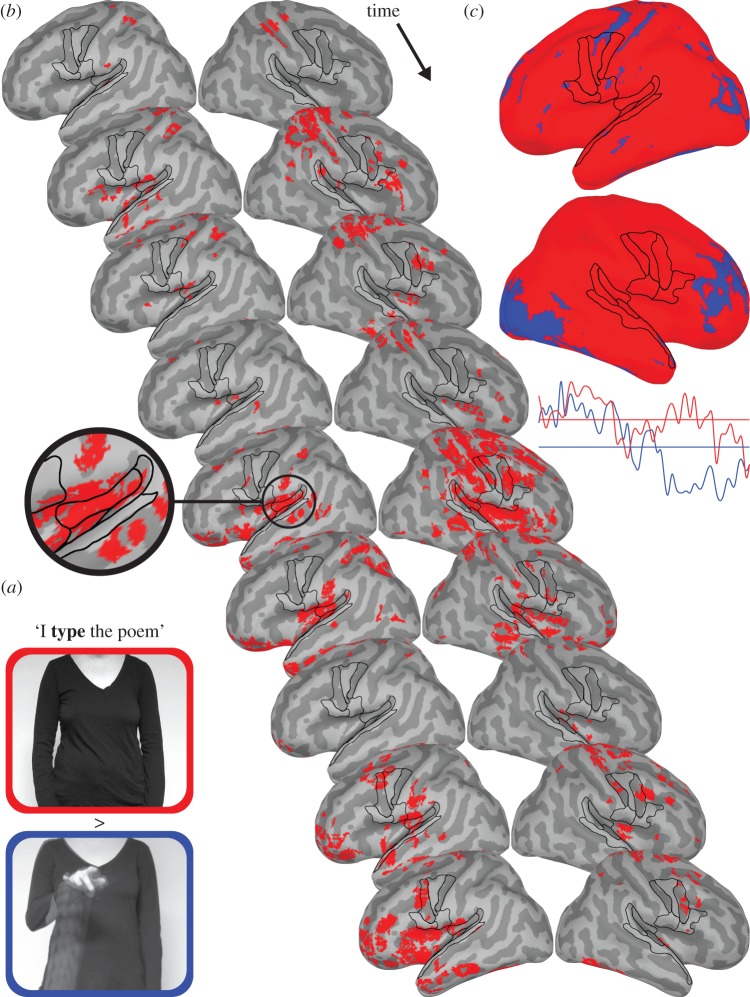
Comparison of lexical affiliates in sentences when preceded by iconic co-speech gestures that visually described those words (blue) and when not preceded by those gestures (red). (*a*) Example frames from ‘I type the poem’ for the no-gesture (top/red) and gesture video clips (bottom/blue). The latter was constructed to show hand and arm motion from the start of the sentence to the end of the lexical affiliate visually described by the gesture (‘type’). (*b*) Beginning 148 ms after the onset of the lexical affiliate and progressing in 4 ms steps to 184 ms, brain images show significant no-gesture > iconic gesture activity. The inset at 164 ms magnifies the primary AC and PST regions (figure 1*a*). Brain images at 180 ms are not shown because there were no significant differences. All *p*'s ≤ 0.05 FDR corrected for multiple comparisons with a cluster size of 20 voxels. (*c*) Brain images show the mean of activity from the onset of the lexical affiliates to 184 ms illustrating more overall activity for the no-gesture condition. Time series below those images are the averaged bilateral primary AC (TTG) response for that time period and the horizontal lines are the means of those time series.

Stimuli were 168 randomly presented video clips (mean length = 4422 ms). There were 56 iconic gesture, 28 filler gesture, 56 iconic no-gesture and 28 filler no-gesture clips. Each video faded in and out for 500 ms. The actress wore plain black clothing and videos were recorded so that her body above the neck and below the waist were not visible (figure 3*a*).

Iconic gesture clips were constructed so that the gestures visually enacted actions associated with lexical affiliates that followed. Examples include typing motions for the lexical affiliate ‘type’ in ‘I type the poem’ or running as portrayed by alternating index and middle finger movements for the lexical affiliate ‘flee’ in ‘I flee the house’. On average, iconic gestures began 713 ms after the start of the clip and the preparation phase of the gesture lasted 392 ms. The meaningful aspect of the gestures (i.e. containing the iconic information), measured from the end of preparation phase, began on average 444 ms before the start of the audio. The lexical affiliate began on average 183 ms following the start of the audio. Therefore, 627 ms of meaningful information was available in the iconic gestures before the lexical affiliate associated with those gestures occurred.

Gestures in filler gesture clips were not as constrained nor as iconic as the gestures in the iconic gesture clips (e.g. a hand being raised skyward for ‘I judge the match’). Filler clips were used to conceal the focus of the experiment on the more meaningful/predictive information in the iconic gestures and were not analysed. The iconic no-gesture and filler no-gesture conditions were created by removing the video from the gesture clips and replacing them with still images of the actress with her arms at her sides. All stimuli were separated by a 100–200 ms randomly varying inter-stimulus interval (ISI). Participants passively viewed stimuli on a 14 inch monitor that was about 2–3 feet in front of them. Clips were presented at fixation at half the screen size to prevent head movements and saccades. Blinks and saccades were tracked with an eye tracker.

#### (iii) Preprocessing

EEG was collected from 256-channels at a sampling rate of 250 Hz (Electrical Geodesics, Inc., http://www.egi.com/). All preprocessing, source localization and data analyses were done with Brainstorm software unless otherwise noted (http://neuroimage.usc.edu/brainstorm) [72]. Ocular artefacts were removed using signal-space projection [73,74] and bad channels were removed. The resulting data were bandpass filtered from 1 to 100 Hz. The data were average re-referenced. Trials were segmented from the start of the lexical affiliate to the end of the audio. Those trials with a peak-to-peak amplitude of more than 150 µV were discarded and all remaining trials were averaged. One-hundred milliseconds of the ISI after each trial was used to compute the noise covariance for source localization. The trials were also *z*-score normalized by the level of noise calculated over the same 100 ms time period. Finally, source localization used the OpenMEEG BEM forward model [75] with the N27 template anatomy and sLORETA inverse model [76] with depth weighting.

#### (iv) Group analysis

Paired *t*-tests were used to compare the lexical affiliate for the iconic gesture and no-gesture conditions. *t*-tests were done in each voxel at each time point from the start of lexical affiliate to the end of the audio. Each whole brain image at each timepoint was FDR corrected for multiple comparisons to *p* = 0.05. A cluster size threshold of 20 voxels was used to further protect against false positives.

### (b) Results

Paired *t*-tests show a time window of more statistically significant activity for the no-gesture condition compared with the gesture condition. Specifically, the no-gesture condition produced significantly more activity from 148 to 176 ms and at 184 ms following onset of the lexical affiliate visually described by the gestures. There was no significantly different activity between conditions at 180 ms. Greater no-gesture activity in this time window, though variable in location, was fairly consistently localized to the PST and PVF regions (figure 3*b* (red)). There were no regions that were significantly more active for the gesture compared with the no-gesture condition, perhaps reflecting the conservative corrected statistical threshold used. Indeed, the mean activity over the temporal window from the onset of the lexical affiliate to 184 ms shows that there are regions that trend towards being consistently more active for the gesture condition. These include, most prominently, regions dorsal to the PVF regions, pre-frontal and visual cortices (figure 3*c* (blue)).

## 4. Discussion

Counterintuitively and contrary to the opinion of most neuroscientists that AC should be more activated by meaningful linguistic stimuli (see the electronic supplementary material, §3), results show that primary AC (i.e. the transverse temporal gyrus, TTG) and other PST regions are strikingly *less* engaged by more meaningful linguistic compared with less meaningful stimuli and tasks. Increased PST engagement by these less meaningful stimuli and tasks co-occurred with more activity in PVF regions and other regions also involved in SP. Less activity for meaningful linguistic and more activity for less meaningful stimuli and tasks in PST and PVF regions are driven by the presence or relative absence of predictive verbal and non-verbal context, respectively. In the following, a detailed summary of the results is given and alternative explanations discussed (§4*a*). After, results are discussed in relation to the model of the natural organization of language and the brain described in the introduction (§4*b*). Finally, more general implications of the results are suggested (§4*c*).

### (a) Summary

The first set of neuroimaging meta-analyses showed that AC was more engaged by a large heterogeneous array of less semantically meaningful stimuli and tasks, i.e. when less predictive context was available (§2*b*). To review, when it was active, primary AC was engaged by auditory stimuli as much as it was by stimuli from other sensory modalities and demonstrated a strong proclivity to be activated more in studies that are in something other than the more meaningful behavioural domain of language. Indeed, more than 3/5 of the studies that reported primary AC activity did not use meaningful linguistic stimuli (§2*b*(i)). When the constraint that activity locations be in primary AC was removed and search criteria narrowed to include only experimental situations that somewhat resembled everyday language use (in that participants only passively listened and did not perform meta-linguistic tasks), PST regions (including primary AC) were more strongly engaged by less meaningful sounds compared with words (§2*b*(ii)). Furthermore, these regions (along with PVF regions) formed a network during passive listening that was overwhelmingly more correlated with less, e.g. non-vocal, compared with more meaningful sounds. Word stimuli were correlated with this passive listening network as much as *no stimulus presentation at all* (§2*b*(ii)). Finally, criteria that stimuli be auditory or that participants not be engaged in meta-linguistic tasks were relaxed and the search criteria expanded to include any studies in the behavioural domain of language and speech, phonology, syntax or semantics. PST regions were again more engaged by experiments described as being in the less (speech and phonology) than more (syntax or semantics) meaningful behavioural domains (§2*b*(iii)). Finally, in addition to being relatively less activated, PST regions were sometimes deactivated by more meaningful linguistic stimuli and tasks (§2*d*(ii)).

The SP meta-analyses results were striking in the seemingly obligatory degree to which brain regions involved in SP were activated with AC when it was engaged by less meaningful stimuli and tasks, i.e. when less predictive context was available (§2*c*). These regions included not only PVF regions but also other ‘core’ SP regions like the anterior insula, SMA, basal ganglia and cerebellum [27,28]. Specifically, when activity locations in the primary AC were reported, they were co-activated, i.e. they formed a network with regions that greatly overlapped with both overt and covert SP. For example, there was a 71% overlap of primary AC co-activity with covert SP across the whole brain. Consistent with other studies [54,55], covert production tasks rule out the possibility that overlap in AC can simply be accounted for by participants hearing their own vocalizations. The intrinsic connectivity network analysis for passive listening (strongly driven by less meaningful stimuli) also showed that primary AC and other PST regions form a functional network with PVF regions (§2*b*(ii)). Similarly, for contrast analyses, passively listening to less meaningful sounds > words and studies in the behavioural domain of speech or phonology > syntax or semantics overlapped *more* with overt and covert SP compared with much less overlap for the converse more meaningful contrasts. Across all analyses, depending on the comparison made, less meaningful stimuli and tasks resulted in three to nine (mean = 6) times more activity overlap with SP than more meaningful stimuli and tasks (table 3).

It might be argued that results of the meta-analyses can be accounted for by some systematic difference across studies in stimuli or tasks that reflect something other than meaningfulness or predictability. This seems unlikely because such differences are typically ‘subtracted out’ and are, therefore, not likely to contribute to activation patterns. Indeed, it would be difficult to publish an experiment in which stimuli are simply compared to a resting baseline or not ‘matched’ in some way (e.g. speech versus revered speech) or in which ‘higher level’ stimuli/control tasks do not account for levels of attention, task difficulty or other differences. Furthermore, some of the meta-analyses themselves suggest that results are not due to systematic differences in stimuli or tasks. The passive listening meta-analysis held stimuli (auditorily presented sounds) and task (i.e. listening) constant throughout and produced similar and overlapping results with task-driven meta-analyses that use a large variety of stimuli and tasks. Similarly, results do not change when meta-analyses that are limited to contrasts with a resting baseline are directly compared, suggesting that results are not due to the comparison conditions themselves.

An experiment directly manipulating context was conducted to provide support for the *hypothesis* that it was the added predictive context in more meaningful stimuli and tasks driving meta-analyses results (§3). Furthermore, the experiment extended results to serve as an example of the use of multimodal non-verbal context by the brain. Specifically, when iconic co-speech gestures that visually described forthcoming lexical affiliates were observed by participants, there was decrease in AC activity when those lexical affiliates occurred compared with the same words when not preceded by gestures. That is, when gestures were *not* observed by participants, there was an increase in PST activity for words compared with when those words were preceded by gestures. This increase in PST activity for lexical affiliates not preceded by gesture was accompanied by a large increase in activity in PVF regions that were also active in the SP meta-analyses.

### (b) The natural organization of language and the brain model

The NOLB model described in §1*b* can account for meta-analyses and experimental results. According to this model, the brain uses context available in naturalistic or real-world conditions to form and test hypotheses, i.e. make predictions about the identity of forthcoming speech sounds. When predictions originating from PVF–SP regions are accurate, no error signal is generated in AC and no more or, at least, less processing is required. There was more linguistic context to derive predictions from in the more meaningful meta-analyses (e.g. phonemes in words versus pseudo-words) and more non-linguistic context to derive predictions from in the experiment (i.e. words preceded by versus not preceded by gestures that describe those words). More accurate predictions could be generated from these verbal and non-verbal types of context, resulting in less error signal and, therefore, less metabolic expenditure in PST, PVF–SP and other regions. Conversely, less accurate predictions would be generated in the absence of those forms of context, resulting in more network interactions between PST, PVF–SP and other regions and, therefore, more metabolic expenditure (see [77,78] for a network analysis of discourse with and without co-speech gestures). In 4*b*(i)(ii), results suggesting that the process of predicting reuse regions involved in SP and that the result is a metabolic saving are expanded upon with respect to the NOLB model.

#### (i) Speech production regions

There has been a resurgence of research and theory pertaining to ‘the predictive brain’ [79]. One review suggests that predictions can come from most regions of the brain [80]. This begs the question as to why in the present results, the active hypothesis-and-test mechanism seems to so strongly rely on a network of regions supporting SP, including PVF regions, the anterior insula, basal ganglia, SMA and cerebellum. Before speculating as to why, it should be noted that this does not imply that predictions are not also generated in other regions of the brain. It should also be noted that the possibility exists that, though SP regions are active, this activity could have nothing to do with the role of those regions in SP. That is, it may be that the computations being performed are unrelated to the computations supporting SP. That caveat aside, there are quite a few reasons why the NOLB model hypothesis-and-test mechanism might ‘neurally re-use’ [25] brain regions supporting SP ([16,17], see also [81]). The most obvious is that speech perception and production both act upon the same auditory objects. Why institute a new set of regions to perform computations for speech perception that are already being performed on those objects during production?

One computation already implemented in production that can be re-used in the process of speech perception is ‘selection’. The SMA has been suggested to play a role in the selection of competing lexical items during SP [82,83]. Thus, this region would seem to be a good candidate for selecting among the various competing auditory objects that would be activated by the association of context and related auditory objects. A second reusable computation is prediction. As discussed (§1*b*(ii)), there is a predictive mechanism already in place in SP, involving PVF regions, for adjusting vocalizations in real time based on efference copy to AC [84–88]. This feedback causes a reduction in AC activity, corresponding to an increase in sensitivity for vocal feedback [31] that is quite specific [88–90]. Re-using this mechanism constitutes a method by which a selected hypothesized auditory object can be pre-activated in AC and provide a constraint on the interpretation of acoustic patterns. A third re-usable computation is sequencing. Language evolves temporally and, therefore, it would seem that these predictions need a way to be kept active (possibly involving articulatory mechanisms, e.g. [91]) and sequenced to at least partially correspond in time with (forthcoming) associated acoustic patterns. The basal ganglia and SMA have been particularly implicated in sequencing during both speech perception and production [92–95], as has the cerebellum [96]. These regions might provide a mechanism for a selected hypothesis to be scheduled for prediction.

There is also a more general argument to be made for why SP regions would be re-used in real-world language use. Specifically, humans have agreed on a set of shared labels, i.e. words for a variety of experiences of the world. These experiences are more variable than (the categorical) words we use to describe those experiences [97]. For example, the sight of a four-legged creature with a tail, the sound of ‘meow’ and feeling of fur are all related to the word ‘cat’. A purely associative process between those (non-linguistic) experiences and related (linguistic) auditory objects would lead to the pre-activation of a fairly unconstrained portion of AC (corresponding to the semantic neighbourhood of ‘cat’). By contrast, being able to re-use existing abilities of the SP system to select, sequence and activate corresponding auditory objects predictively could lead to the *specific* pre-activation of /k/ or ‘cat’ in AC and constrain interpretation of auditory patterns in a less variable way. In summary, there are a set of computations, i.e. selection, prediction and sequencing, that are likely computed during SP, which could be re-used during speech perception. This would help constrain the problem of the non-deterministic mapping between what arrives in AC and what we hear.

#### (ii) Metabolic savings

Results suggest that context is used to generate predictions and that this process ultimately results in a decrease in metabolic expenditure both in terms of relative activation and, possibly, deactivation (see [98–100] for other examples of language- and auditory-related deactivation). These metabolic savings may be an underestimation when considering that speech encountered during everyday language use co-occurs with far more context with which to make predictions. For example, co-speech gestures were available in the experiment but most common forms of natural context, e.g. a larger discourse context and observable speech-associated mouth movements, were absent. Nonetheless, the mean of activity associated with the lexical affiliates of the gestures suggests that most of the brain is less active on average compared with when even this minimal context is missing (figure 3*c*). Thus, the predictive mechanism supported by the results, though seemingly expensive to implement, would ultimately conserve a great deal of metabolic resources out in the real world. It is an open question as to whether these savings are more than the metabolic demands required to implement the active mechanism or that might be required by a more feed-forward model that does not actively use context. This latter point is somewhat irrelevant, however, as the active hypothesis-and-test mechanism is argued to be *necessary* for speech perception to occur to achieve perceptual constancy (§1*b*(i), see also [101,102]) and may also be necessary for conversations to occur as they do. That is, it would be hard to explain the median 120 ms gap between conversational turns [103] with something other than an active/predictive model [104].

A reduction in activity like that seen here is not without precedence. For example, when there has been a theoretical motivation to examine less meaningful sounds, AC activity has been reported to be more active for less meaningful sounds than speech [105–107]. Otherwise, the bulk of the supporting data requires that one reinterpret other findings. In particular, there are several ubiquitous effects that are contingent on repeated and surprising stimulus presentations that Friston and co-workers have suggested be re-characterized in a predictive coding (free-energy) framework, including repetition suppression (RS) and the mismatch negativity (MMN) [108]. In the RS case, stimuli are more predictable because they are repeating and this leads to less prediction error and, therefore, less AC activity [108–111]. In the MMN case, repeating stimuli similarly lead to less AC activity and the surprising event, because it is not predictable, results in a large prediction error in AC and, therefore, more activity relative to the repeating event [108,112,113].

There is also quite a bit of electrophysiology work consistent with the present results. The MMN has been likened to findings in the cat, in which AC response is increased to rarely presented sounds compared with the same sounds when commonly presented [114]. In summarizing this and other work, the author says that ‘A1 neurons are exquisitely suitable for novelty detection and for change detection … the unique function of A1 is not in feature detection’ [115, p. VII]. Related conclusions have been drawn from findings in other animals. For example, using Fos imaging, 1.4 times more neurons were labelled by novel compared with familiar sounds in rat AC [116]. In the bird, at least some parts of AC encode ‘surprise’ and not sound intensity variations. For example, AC fires more during an unexpected *silence* and the AC response when played random noise is as if the birds ‘were expecting conspecific song, finding the inconsistencies between birdsong and noise surprising’ [117].

### (c) Implications

Collectively, these results serve as a cautionary tale with regard to thinking about hearing and AC functioning from our phenomenological experience and folk psychological beliefs about the brain. That is, the results lead to quite different and unintuitive answers to the questions of what we hear and what AC does with speech from those hypothetically given at the outset of the introduction. Specifically, rather than ‘sounds’, results suggest that what we hear is mostly internally generated and in the form of ‘unconscious inferences’ about the identity of upcoming sound patterns. These inferences or hypotheses derive from the association of context in our linguistic environment with our internal knowledge of the world. That knowledge ultimately takes the form of predictions from motor regions involved in SP and, therefore, the ‘sounds’ we hear are in some large part actually ‘echoes of the spoken past’. The role of AC is to confirm or deny those predictions based on impoverished evidence from the auditory world. AC, therefore, is often the end rather than the beginning of a processing hierarchy leading to language comprehension. This characterization is more consistent with data reviewed in the Introduction suggesting that AC is an ‘auditory object’ processor characterized by a great many feedback connections from other cortical regions.

Results also imply that we must begin to take context more seriously and acknowledge that hearing speech is deeply multimodal and that any strong separation between ‘verbal’ and ‘non-verbal’ is a false distinction. This is because any form of context can be used, through associative processes, to generate and test hypotheses about the nature of auditory information through prediction. Thus, external visual, somatosensory and olfactory context and internal context can all ultimately be associated with auditory objects (or vice versa). Thus, for example, participants in the co-speech gesture experiment were really ‘hearing’ the visual non-verbal gestures in some way because those movements were mapped onto related words and used to predict corresponding acoustic objects in AC. Perhaps AC did not demonstrate a strong preference for any one sensory modality in the analyses done in this manuscript (§2*b*(i)) because it is activated in a mostly feedback manner, potentially originating from any number of other sensory modalities associated with available context.

Finally, results suggest that we take more seriously the proposal that hearing is an active or constructive process primarily characterized by, if anything, feedback in the brain. This has strong implications for how we think about the role of AC in disorders of language. Consistent with an active feedback model, it has been shown that it is auditory efferent connections, e.g. from the planum temporale to the TTG, that determine how severe speech processing deficits are following stroke [118,119]. Likewise, both the auditory hallucinations accompanying schizophrenia [87,120–122] and stuttering [27] have been associated with disorders of efference copy from frontal SP regions to AC. Similarly, AC processing deficits in autism [123] might well be associated with poor long-range connectivity from frontal SP to AC regions [124]. Re-conceptualizing speech perception and the role of AC in hearing speech as an active (mostly) feedback process that is reliant on context might help us gain some traction in understanding these disorders.

## Supplementary Material

Supplementary material

## References

[1] Cordemoy GD 1668 A philosophical discourse concerning speech, conformable to the Cartesian principles. In the Savoy, London.

[2] Emerson RW 1856 English traits. London, UK: G. Routledge and Co.

[3] Pennebaker JW 1997 Opening up: the healing power of expressing emotions. New York, NY: The Guilford Press, rep sub edition edn.

[4] Wang X 2007 Neural coding strategies in auditory cortex. Hear. Res. 229, 81–93. (10.1016/j.heares.2007.01.019) 17346911

[5] Nelken I 2004 Processing of complex stimuli and natural scenes in the auditory cortex. Curr. Opin. Neurobiol. 14, 474–480. (10.1016/j.conb.2004.06.005) 15321068

[6] Winer JA Miller LM Lee CC Schreiner CE 2005 Auditory thalamocortical transformation: structure and function. Trends Neurosci. 28, 255–263. (10.1016/j.tins.2005.03.009) 15866200

[7] Nudds M 2010 What are auditory objects? Rev. Phil. Psychol. 1, 105–122. (10.1007/s13164-009-0003-6)

[8] Scott SK 2005 Auditory processing: speech, space and auditory objects. Curr. Opin. Neurobiol. 15, 197–201. (10.1016/j.conb.2005.03.009) 15831402

[9] Kayser C Petkov CI Remedios R Logothetis NK 2012 Multisensory influences on auditory processing: perspectives from fMRI and electrophysiology. In The neural bases of multisensory processes (eds Murray MM Wallace MT), pp. 99–114. Frontiers in Neuroscience Boca Raton, FL: CRC Press. 22593869

[10] Nelken I 2008 Processing of complex sounds in the auditory system. Curr. Opin. Neurobiol. 18, 413–417. (10.1016/j.conb.2008.08.014) 18805485

[11] Nelken I Bar-Yosef O 2008 Neurons and objects: the case of auditory cortex. Front. Neurosci. 2, 107 (10.3389/neuro.01.009.2008) 18982113 PMC2570071

[12] Wang X Lu T Bendor D Bartlett E 2008 Neural coding of temporal information in auditory thalamus and cortex. Neuroscience 154, 294–303. (10.1016/j.neuroscience.2008.03.065) 18555164 PMC2751884

[13] Budinger E Scheich H 2009 Anatomical connections suitable for the direct processing of neuronal information of different modalities via the rodent primary auditory cortex. Hear. Res. 258, 16–27. (10.1016/j.heares.2009.04.021) 19446016

[14] Scheich H Brechmann A Brosch M Budinger E Ohl FW 2007 The cognitive auditory cortex: task-specificity of stimulus representations. Hear. Res. 229, 213–224. (10.1016/j.heares.2007.01.025) 17368987

[15] Winer JA 2011 A profile of auditory forebrain connections and circuits. In The auditory cortex (eds Winer JA Schreiner CE), pp. 41–74. New York, NY: Springer.

[16] Skipper JI In press. The NOLB model: a model of the natural organization of language and the brain. In Cognitive neuroscience of natural language use (ed. Roel Willems) Cambridge, UK: Cambridge University Press.

[17] Skipper JI Nusbaum HC Small SL 2006 Lending a helping hand to hearing: another motor theory of speech perception. In Action to language via the mirror neuron system (ed. Arbib MA), pp. 250–286. Cambridge, MA: Cambridge University Press.

[18] Callan D Callan A Gamez M Sato M-A Kawato M 2010 Premotor cortex mediates perceptual performance. NeuroImage 51, 844–858. (10.1016/j.neuroimage.2010.02.027) 20184959

[19] Callan DE Jones JA Callan AM Akahane-Yamada R 2004 Phonetic perceptual identification by native- and second-language speakers differentially activates brain regions involved with acoustic phonetic processing and those involved with articulatory–auditory/orosensory internal models. NeuroImage 22, 1182–1194. (10.1016/j.neuroimage.2004.03.006) 15219590

[20] Poeppel D Idsardi WJ van Wassenhove V 2008 Speech perception at the interface of neurobiology and linguistics. Phil. Trans. R. Soc. B 363, 1071–1086. (10.1098/rstb.2007.2160) 17890189 PMC2606797

[21] Rauschecker JP Scott SK 2009 Maps and streams in the auditory cortex: nonhuman primates illuminate human speech processing. Nat. Neurosci. 12, 718–724. (10.1038/nn.2331) 19471271 PMC2846110

[22] Appelbaum I 1996 The lack of invariance problem and the goal of speech perception. In Fourth International Conf. on Spoken Language Processing, pp. 1541–1544. See http://www.asel.udel.edu/icslp/cdrom/vol3/435/a435.pdf.

[23] Gregory RL 1997 Knowledge in perception and illusion. Phil. Trans. R. Soc. Lond. B 352, 1121–1127. (10.1098/rstb.1997.0095) 9304679 PMC1692018

[24] Pulvermüller F 1999 Words in the brain's language. Behav. Brain Sci. 22, 253–279. (10.1017/S0140525X9900182X) 11301524

[25] Anderson ML 2010 Neural reuse: a fundamental organizational principle of the brain. Behav. Brain Sci. 33, 245–266. (10.1017/S0140525X10000853) 20964882

[26] Tschida K Mooney R 2012 The role of auditory feedback in vocal learning and maintenance. Curr. Opin. Neurobiol. 22, 320–327. (10.1016/j.conb.2011.11.006) 22137567 PMC3297733

[27] Brown S Ingham RJ Ingham JC Laird AR Fox PT 2005 Stuttered and fluent speech production: an ALE meta-analysis of functional neuroimaging studies. Hum. Brain Mapp. 25, 105–117. (10.1002/hbm.20140) 15846815 PMC6871755

[28] Eickhoff SB Heim S Zilles K Amunts K 2009 A systems perspective on the effective connectivity of overt speech production. Phil. Trans. R. Soc. A 367, 2399–2421. (10.1098/rsta.2008.0287) 19414462 PMC3268212

[29] Grabski K Lamalle L Vilain C Schwartz J Vallée N Tropres I Baciu M Le Bas J Sato M 2012 Functional MRI assessment of orofacial articulators: neural correlates of lip, jaw, larynx, and tongue movements. Hum. Brain Mapp. 33, 2306–2321. (10.1002/hbm.21363) 21826760 PMC6870116

[30] Takai O Brown S Liotti M 2010 Representation of the speech effectors in the human motor cortex: Somatotopy or overlap? Brain Lang. 113, 39–44. (10.1016/j.bandl.2010.01.008) 20171727

[31] Eliades SJ Wang X 2008 Neural substrates of vocalization feedback monitoring in primate auditory cortex. Nature 453, 1102–1106. (10.1038/nature06910) 18454135

[32] Eliades SJ Wang X 2013 Comparison of auditory–vocal interactions across multiple types of vocalizations in marmoset auditory cortex. J. Neurophysiol. 109, 1638–1657. (10.1152/jn.00698.2012) 23274315 PMC3602939

[33] Sumby WH Pollack I 1954 Visual contribution to speech intelligibility in noise. J. Acoust. Soc. Am. 26, 212 (10.1121/1.1907309)

[34] Chandrasekaran C Trubanova A Stillittano S Caplier A Ghazanfar AA 2009 The natural statistics of audiovisual speech. PLoS Comput. Biol. 5, e1000 436 (10.1371/journal.pcbi.1000436) PMC2700967 19609344

[35] Skipper JI Nusbaum HC Small SL 2005 Listening to talking faces: motor cortical activation during speech perception. Neuroimage 25, 76–89. (10.1016/j.neuroimage.2004.11.006) 15734345

[36] Skipper JI van Wassenhove V Nusbaum HC Small SL 2007 Hearing lips and seeing voices: how cortical areas supporting speech production mediate audiovisual speech perception. Cereb. Cortex 17, 2387–2399. (10.1093/cercor/bhl147) 17218482 PMC2896890

[37] Hasson U Skipper JI Nusbaum HC Small SL 2007 Abstract coding of audiovisual speech: beyond sensory representation. Neuron 56, 1116–1126. (10.1016/j.neuron.2007.09.037) 18093531 PMC2175551

[38] Jones JA Callan DE 2003 Brain activity during audiovisual speech perception: an fMRI study of the McGurk effect. Neuroreport 14, 1129–1133. (10.1097/00001756-200306110-00006) 12821795

[39] Szycik GR Stadler J Tempelmann C Münte TF 2012 Examining the McGurk illusion using high-field 7 tesla functional MRI. Front. Hum. Neurosci. 6, 95 (10.3389/fnhum.2012.00095) 22529797 PMC3329794

[40] Sato M Cavé C Ménard L Brasseur A 2010 Auditory-tactile speech perception in congenitally blind and sighted adults. Neuropsychologia 48, 3683–3686. (10.1016/j.neuropsychologia.2010.08.017) 20736028

[41] Arnal LH Wyart V Giraud A 2011 Transitions in neural oscillations reflect prediction errors generated in audiovisual speech. Nat. Neurosci. 14, 797–801. (10.1038/nn.2810) 21552273

[42] Kauramäki J Jääskeläinen IP Hari R Möttönen R Rauschecker JP Sams M 2010 Lipreading and covert speech production similarly modulate human auditory-cortex responses to pure tones. J. Neurosci. 30, 1314–1321. (10.1523/JNEUROSCI.1950-09.2010) 20107058 PMC2832801

[43] van Wassenhove V Grant KW Poeppel D 2005 Visual speech speeds up the neural processing of auditory speech. Proc. Natl Acad. Sci. USA 102, 1181–1186. (10.1073/pnas.0408949102) 15647358 PMC545853

[44] Fox PT Laird AR Fox SP Fox PM Uecker AM Crank M Koenig SF Lancaster JL 2005 BrainMap taxonomy of experimental design: description and evaluation. Hum. Brain Mapp. 25, 185–198. (10.1002/hbm.20141) 15846810 PMC6871758

[45] Fox PT Lancaster JL 2002 Mapping context and content: the brain-map model. Nat. Rev. Neurosci. 3, 319–321. (10.1038/nrn789) 11967563

[46] Laird AR Lancaster JL Fox PT 2005 BrainMap: the social evolution of a human brain mapping database. Neuroinformatics 3, 65–78. (10.1385/NI:3:1:065) 15897617

[47] Laird AR 2010 Comparison of the disparity between talairach and MNI coordinates in functional neuroimaging data: validation of the Lancaster transform. NeuroImage 51, 677–683. (10.1016/j.neuroimage.2010.02.048) 20197097 PMC2856713

[48] Lancaster JL Tordesillas-Gutiérrez D Martinez M Salinas F Evans A Zilles K Mazziotta JC Fox PT 2007 Bias between MNI and talairach coordinates analyzed using the ICBM-152 brain template. Hum. Brain Mapp. 28, 1194–1205. (10.1002/hbm.20345) 17266101 PMC6871323

[49] Eickhoff SB Bzdok D Laird AR Kurth F Fox PT 2012 Activation likelihood estimation meta-analysis revisited. Neuroimage 59, 2349–2361. (10.1016/j.neuroimage.2011.09.017) 21963913 PMC3254820

[50] Eickhoff SB Bzdok D Laird AR Roski C Caspers S Zilles K Fox PT 2011 Co-activation patterns distinguish cortical modules, their connectivity and functional differentiation. Neuroimage 57, 938–949. (10.1016/j.neuroimage.2011.05.021) 21609770 PMC3129435

[51] Turkeltaub PE Eickhoff SB Laird AR Fox M Wiener M Fox P 2012 Minimizing within-experiment and within-group effects in activation likelihood estimation meta-analyses. Hum. Brain Mapp. 33, 1–13. (10.1002/hbm.21186) 21305667 PMC4791073

[52] Laird AR 2011 Behavioral interpretations of intrinsic connectivity networks. J. Cogn. Neurosci. 23, 4022–4037. (10.1162/jocn_a_00077) 21671731 PMC3690655

[53] Diedenhofen B 2013 cocor: Comparing correlations. See http://birkdiedenhofen.de/R-repo/pckg/cocor/.

[54] Grabski K Lamalle L Sato M 2012 Somatosensory-motor adaptation of orofacial actions in posterior parietal and ventral premotor cortices. PLoS ONE 7, e49117 (10.1371/journal.pone.0049117) 23185300 PMC3502466

[55] Shergill SS Brammer MJ Fukuda R Bullmore E Amaro E Murray RM McGuire PK 2002 Modulation of activity in temporal cortex during generation of inner speech. Hum. Brain Mapp. 16, 219–227. (10.1002/hbm.10046) 12112764 PMC6871832

[56] Mangia S Giove F Tkáč I Logothetis NK Henry P Olman CA Maraviglia B Di Salle F Uğurbil K 2009 Metabolic and hemodynamic events after changes in neuronal activity: current hypotheses, theoretical predictions and *in vivo* NMR experimental findings. J. Cereb. Blood Flow Metab. 29, 441–463. (10.1038/jcbfm.2008.134) 19002199 PMC2743443

[57] Laurienti PJ Burdette JH Wallace MT Yen Y Field AS Stein BE 2002 Deactivation of sensory-specific cortex by cross-modal stimuli. J. Cogn. Neurosci. 14, 420–429. (10.1162/089892902317361930) 11970801

[58] Wise R Greene J Büchel C Scott S 1999 Brain regions involved in articulation. Lancet 353, 1057–1061. (10.1016/S0140-6736(98)07491-1) 10199354

[59] McNeill D 1992 Hand and mind: what gestures reveal about thought. Chicago, IL: University of Chicago Press.

[60] Hostetter AB 2011 When do gestures communicate? A meta-analysis. Psychol. Bull. 137, 297–315. (10.1037/a0022128) 21355631

[61] Goldin-Meadow S Alibali MW 2013 Gesture's role in speaking, learning, and creating language. Annu. Rev. Psychol. 64, 257–283. (10.1146/annurev-psych-113011-143802) 22830562 PMC3642279

[62] Bergmann K Aksu V Kopp S 2011 The relation of speech and gestures: temporal synchrony follows semantic synchrony. *Proc. 2nd Workshop on Gesture and Speech in Interaction* (*GeSpIn 2011*), *Bielefeld, Germany, 5–7 Sep 2011*. See http://www.techfak.uni-bielefeld.de/~kbergman/download/Bergmann+Aksu+Kopp2011.pdf.

[63] Morrel-Samuels P Krauss RM 1992 Word familiarity predicts temporal asynchrony of hand gestures and speech. J. Exp. Psychol. Learn. Mem. Cogn. 18, 615 (10.1037/0278-7393.18.3.615)

[64] Bernardis P Salillas E Caramelli N 2008 Behavioural and neurophysiological evidence of semantic interaction between iconic gestures and words. Cogn. Neuropsychol. 25, 1114–1128. (10.1080/02643290801921707) 18608334

[65] Kelly SD Ozyurek A Maris E 2010 Two sides of the same coin: speech and gesture mutually interact to enhance comprehension. Psychol. Sci. 21, 260–267. (10.1177/0956797609357327) 20424055

[66] So W Yi-Feng AL Yap D Kheng E Yap JM 2013 Iconic gestures prime words: comparison of priming effects when gestures are presented alone and when they are accompanying speech. Front. Psychol. 4, 779 (10.3389/fpsyg.2013.00779) 24155738 PMC3800814

[67] Wu YC Coulson S 2007 Iconic gestures prime related concepts: an ERP study. Psychon. Bull. Rev. 14, 57–63. (10.3758/BF03194028) 17546731

[68] Yap D So W Melvin Yap J Tan Y Teoh RS 2011 Iconic gestures prime words. Cogn. Sci. 35, 171–183. (10.1111/j.1551-6709.2010.01141.x) 21428996

[69] Glover GH 1999 Deconvolution of impulse response in event-related BOLD fMRI. NeuroImage 9, 416–429. (10.1006/nimg.1998.0419) 10191170

[70] Im C Gururajan A Zhang N Chen W He B 2007 Spatial resolution of EEG cortical source imaging revealed by localization of retinotopic organization in human primary visual cortex. J. Neurosci. Methods 161, 142–154. (10.1016/j.jneumeth.2006.10.008) 17098289 PMC1851670

[71] Oldfield RC 1971 The assessment and analysis of handedness: the Edinburgh inventory. Neuropsychologia 9, 97–113. (10.1016/0028-3932(71)90067-4) 5146491

[72] Tadel F Baillet S Mosher JC Pantazis D Leahy RM 2011 Brainstorm: a user-friendly application for MEG/EEG analysis. Comput. Intell. Neurosci. 2011, 1–13. (10.1155/2011/879716) 21584256 PMC3090754

[73] Tesche CD Uusitalo MA Ilmoniemi RJ Huotilainen M Kajola M Salonen O 1995 Signal-space projections of MEG data characterize both distributed and well-localized neuronal sources. Electroencephalogr. Clin. Neurophysiol. 95, 189–200. (10.1016/0013-4694(95)00064-6) 7555909

[74] Uusitalo MA Ilmoniemi RJ 1997 Signal-space projection method for separating MEG or EEG into components. Med. Biol. Eng. Comput. 35, 135–140. (10.1007/BF02534144) 9136207

[75] Gramfort A Papadopoulo T Olivi E Clerc M 2010 OpenMEEG: opensource software for quasistatic bioelectromagnetics. Biomed. Eng. Online 9, 45 (10.1186/1475-925X-9-45) 20819204 PMC2949879

[76] Pascual-Marqui RD 2002 Standardized low-resolution brain electromagnetic tomography (sLORETA): technical details. Methods Find. Exp. Clin. Pharmacol. 24 (Suppl. D), 5–12. 12575463

[77] Skipper JI Goldin-Meadow S Nusbaum HC Small SL 2007 Speech-associated gestures, broca's area, and the human mirror system. Brain Lang. 101, 260–277. (10.1016/j.bandl.2007.02.008) 17533001 PMC2703472

[78] Skipper JI Goldin-Meadow S Nusbaum HC Small SL 2009 Gestures orchestrate brain networks for language understanding. Curr. Biol. 19, 661–667. (10.1016/j.cub.2009.02.051) 19327997 PMC3767135

[79] Clark A 2012 Whatever next? Predictive brains, situated agents, and the future of cognitive science. Behav. Brain Sci. 36, 181–204. (10.1017/S0140525X12000477) 23663408

[80] Bubic A Von Cramon DY Schubotz RI 2010 Prediction, cognition and the brain. Front. Hum. Neurosci. 4, 25 (10.3389/fnhum.2010.00025) 20631856 PMC2904053

[81] Pickering MJ Garrod S 2013 An integrated theory of language production and comprehension. Behav. Brain Sci. 36, 329–347. (10.1017/S0140525X12001495) 23789620

[82] Alario F Chainay H Lehericy S Cohen L 2006 The role of the supplementary motor area (SMA) in word production. Brain Res. 1076, 129–143. (10.1016/j.brainres.2005.11.104) 16480694

[83] Piai V Roelofs A Jensen O Schoffelen J Bonnefond M 2014 Distinct patterns of brain activity characterise lexical activation and competition in spoken word production. PLoS ONE 9, e88674 (10.1371/journal.pone.0088674) 24558410 PMC3928283

[84] Guenther FH Ghosh SS Tourville JA 2006 Neural modeling and imaging of the cortical interactions underlying syllable production. Brain Lang. 96, 280–301. (10.1016/j.bandl.2005.06.001) 16040108 PMC1473986

[85] Guenther FH Perkell JS 2004 A neural model of speech production and its application to studies of the role of auditory feedback in speech. In Speech motor control in normal and disordered speech (eds Maassen B Kent R Peters HFM Van Lieshout P Hulstijn W), pp. 29–49. Oxford, UK: Oxford University Press.

[86] Liu H Behroozmand R Larson CR Brudzynski SM 2009 Audiovocal interactions in the mammalian brain. Handb. Mammal. Vocalization 19, 393–402. (10.1016/B978-0-12-374593-4.00036-X)

[87] Pynn LK DeSouza JF 2013 The function of efference copy signals: implications for symptoms of schizophrenia. Vis. Res. 76, 124–133. (10.1016/j.visres.2012.10.019) 23159418

[88] Tian X Poeppel D 2013 The effect of imagination on stimulation: the functional specificity of efference copies in speech processing J. Cogn. Neurosci. 25, 1020–1036. (10.1162/jocn_a_00381) 23469885

[89] Christoffels IK van de Ven V Waldorp LJ Formisano E Schiller NO 2011 The sensory consequences of speaking: parametric neural cancellation during speech in auditory cortex. PLoS ONE 6, e18307 (10.1371/journal.pone.0018307) 21625532 PMC3098236

[90] Niziolek CA Nagarajan SS Houde JF 2013 What does motor efference copy represent? Evidence from speech production. J. Neurosci. 33, 16 110–16 116. (10.1523/JNEUROSCI.2137-13.2013) PMC3792453 24107944

[91] Baddeley A 2003 Working memory: looking back and looking forward. Nat. Rev. Neurosci. 4, 829–839. (10.1038/nrn1201) 14523382

[92] Bohland JW Guenther FH 2006 An fMRI investigation of syllable sequence production. NeuroImage 32, 821–841. (10.1016/j.neuroimage.2006.04.173) 16730195

[93] Kotz SA Schwartze M Schmidt-Kassow M 2009 Non-motor basal ganglia functions: a review and proposal for a model of sensory predictability in auditory language perception. Cortex 45, 982–990. (10.1016/j.cortex.2009.02.010) 19361785

[94] Lieberman P 2002 On the nature and evolution of the neural bases of human language. Am. J. Phys. Anthropol. 119, 36–62. (10.1002/ajpa.10171) 12653308

[95] Tanji J 2001 Sequential organization of multiple movements: involvement of cortical motor areas. Annu. Rev. Neurosci. 24, 631–651. (10.1146/annurev.neuro.24.1.631) 11520914

[96] Ackermann H 2008 Cerebellar contributions to speech production and speech perception: psycholinguistic and neurobiological perspectives. Trends Neurosci. 31, 265–272. (10.1016/j.tins.2008.02.011) 18471906

[97] Lupyan G 2012 What do words do? Toward a theory of language-augmented thought. In Psychology of learning and motivation, vol. 57, pp. 255–297. Elsevier See http://www.sciencedirect.com/science/article/pii/B9780123942937000078.

[98] Azulay H Striem E Amedi A 2009 Negative BOLD in sensory cortices during verbal memory: a component in generating internal representations? Brain Topogr. 21, 221–231. (10.1007/s10548-009-0089-2) 19326203

[99] Linke AC Vicente-Grabovetsky A Cusack R 2011 Stimulus-specific suppression preserves information in auditory short-term memory. Proc. Natl Acad. Sci. USA 108, 12 961–12 966. (10.1073/pnas.1102118108) 21768383 PMC3150893

[100] Shulman GL Fiez JA Corbetta M Buckner RL Miezin FM Raichle ME Petersen SE 1997 Common blood flow changes across visual tasks: II. Decreases in cerebral cortex. J. Cogn. Neurosci. 9, 648–663. (10.1162/jocn.1997.9.5.648) 23965122

[101] Magnuson JS Nusbaum HC 2007 Acoustic differences, listener expectations, and the perceptual accommodation of talker variability. J. Exp. Psychol. Hum. Percept. Perform. 33, 391–409. (10.1037/0096-1523.33.2.391) 17469975

[102] Nusbaum HC Magnuson JS 1997 Talker normalization: phonetic constancy as a cognitive process. In Talker variability in speech processing (eds Johnson KA Mullennix JW), pp. 109–132. New York, NY: Academic Press.

[103] Heldner M Edlund J 2010 Pauses, gaps and overlaps in conversations. J. Phon. 38, 555–568. (10.1016/j.wocn.2010.08.002)

[104] Scott SK McGettigan C Eisner F 2009 A little more conversation, a little less action: candidate roles for the motor cortex in speech perception. Nat. Rev. Neurosci. 10, 295–302. (10.1038/nrn2603) 19277052 PMC4238059

[105] Benson RR Whalen D Richardson M Swainson B Clark VP Lai S Liberman AM 2001 Parametrically dissociating speech and nonspeech perception in the brain using fMRI. Brain Lang. 78, 364–396. (10.1006/brln.2001.2484) 11703063

[106] Binder JR Swanson SJ Hammeke TA Sabsevitz DS 2008 A comparison of five fMRI protocols for mapping speech comprehension systems. Epilepsia 49, 1980–1997. (10.1111/j.1528-1167.2008.01683.x) 18513352 PMC2645716

[107] Whalen DH 2006 Differentiation of speech and nonspeech processing within primary auditory cortex. J. Acoust. Soc. Am. 119, 575 (10.1121/1.2139627) 16454311

[108] Friston K 2005 A theory of cortical responses. Phil. Trans. R. Soc. B 360, 815–836. (10.1098/rstb.2005.1622) 15937014 PMC1569488

[109] Baldeweg T 2006 Repetition effects to sounds: evidence for predictive coding in the auditory system. Trends Cogn. Sci. 10, 93–94. (10.1016/j.tics.2006.01.010) 16460994

[110] Garrido MI Kilner JM Kiebel SJ Stephan KE Baldeweg T Friston KJ 2009 Repetition suppression and plasticity in the human brain. NeuroImage 48, 269–279. (10.1016/j.neuroimage.2009.06.034) 19540921 PMC2821573

[111] Todorovic A van Ede F Maris E de Lange FP 2011 Prior expectation mediates neural adaptation to repeated sounds in the auditory cortex: an MEG study. J. Neurosci. 31, 9118–9123. (10.1523/JNEUROSCI.1425-11.2011) 21697363 PMC6623501

[112] Garrido MI Kilner JM Stephan KE Friston KJ 2009 The mismatch negativity: a review of underlying mechanisms. Clin. Neurophysiol. 120, 453–463. (10.1016/j.clinph.2008.11.029) 19181570 PMC2671031

[113] Zevin JD Yang J Skipper JI McCandliss BD 2010 Domain general change detection accounts for ‘dishabituation’ effects in temporal-parietal regions in functional magnetic resonance imaging studies of speech perception. J. Neurosci. 30, 1110–1117. (10.1523/JNEUROSCI.4599-09.2010) 20089919 PMC2848500

[114] Ulanovsky N Las L Nelken I 2003 Processing of low-probability sounds by cortical neurons. Nat. Neurosci. 6, 391–398. (10.1038/nn1032) 12652303

[115] Ulanovsky N 2004 Neuronal adaptation in cat auditory cortex. PhD thesis The Hebrew University, Jerusalem, Israel.

[116] Wan H Warburton EC Kuśmierek P Aggleton JP Kowalska DM Brown MW 2001 Fos imaging reveals differential neuronal activation of areas of rat temporal cortex by novel and familiar sounds. Eur. J. Neurosci. 14, 118–124. (10.1046/j.0953-816x.2001.01625.x) 11488955

[117] Gill P Woolley SMN Fremouw T Theunissen FE 2008 What's that sound? Auditory area CLM encodes stimulus surprise, not intensity or intensity changes. J. Neurophysiol. 99, 2809–2820. (10.1152/jn.01270.2007) 18287545

[118] Schofield TM Penny WD Stephan KE Crinion JT Thompson AJ Price CJ Leff AP 2012 Changes in auditory feedback connections determine the severity of speech processing deficits after stroke. J. Neurosci. 32, 4260–4270. (10.1523/JNEUROSCI.4670-11.2012) 22442088 PMC3319058

[119] Teki S Barnes GR Penny WD Iverson P Woodhead ZVJ Griffiths TD Leff AP 2013 The right hemisphere supports but does not replace left hemisphere auditory function in patients with persisting aphasia. Brain 136, 1901–1912. (10.1093/brain/awt087) 23715097 PMC3673464

[120] Frith C 2005 The neural basis of hallucinations and delusions. CR Biol. 328, 169–175. (10.1016/j.crvi.2004.10.012) 15771003

[121] Jardri R Pouchet A Pins D Thomas P 2011 Cortical activations during auditory verbal hallucinations in schizophrenia: a coordinate-based meta-analysis. Am. J. Psych. 168, 73–81. (10.1176/appi.ajp.2010.09101522) 20952459

[122] Sommer IEC 2008 Auditory verbal hallucinations predominantly activate the right inferior frontal area. Brain 131, 3169–3177. (10.1093/brain/awn251) 18854323

[123] Boddaert N Belin P Chabane N Poline J Barthélémy C Mouren-Simeoni M Brunelle F Samson Y 2003 Perception of complex sounds: abnormal pattern of cortical activation in autism. Am. J. Psych. 160, 2057–2060. (10.1176/appi.ajp.160.11.2057) 14594758

[124] Travers BG 2012 Diffusion tensor imaging in autism spectrum disorder: a review. Autism Res. 5, 289–313. (10.1002/aur.1243) 22786754 PMC3474893

