# Supplementary material for: Echoes of the spoken past: how auditory cortex hears context during speech perception
Source: Philos Trans R Soc Lond B Biol Sci. 2014 Sep 19;369(1651):20130297. doi: 10.1098/rstb.2013.0297 (PMC4123676; doi:10.1098/rstb.2013.0297)
Supplement: Supplementary material [file rstb20130297supp1.pdf]

Electronic Supplementary Material (ESM) for DOI: 10.1098/rstb.2013.0297:

Echoes of the spoken past: How auditory cortex hears context during speech perception

By Jeremy I. Skipper

Department of Cognitive, Perceptual and Brain Sciences, Institute for Multimodal Communication, University College London, London, UK

ESM A.

1. Parcellation of brain into regions

ALE meta-analyses results were aligned to a surface based version of the MNI N27 anatomical dataset [4] using tools available in AFNI and SUMA (`http://afni.nimh.nih.gov/`)[1, 5]. Cortical reconstruction of surfaces was done with Freesurfer software (`http://surfer.nmr.mgh.harvard.edu/`)[3]. Freesurfer was also used to parcellate the brain into anatomical regions based on gyral and sulcal structure [2]. To make comparisons across meta-analyses easier, all volumes of activation are reported in table 1 using this parcellation scheme. The table excludes activity in white matter and most medial, occipital and subcortical regions. This was done for brevity and because there were no explicit hypotheses about activity in these regions. A horizontal plane placed at the superior most aspect of the inferior precentral sulcus in the Freesurfer parcellation scheme was used as the division between the ventral and dorsal parts of the precentral gyrus and central sulcus in table 1.

2. Additional neuroimaging meta-analyses methods and results

(*a*) *Common search*

All BrainMap database searches were required to meet a set of common search criteria to be included in analyses. These were (in pseudocode where "&" = "and" and "|" = "or"):

> [Experiments, Context, is, Normal Mapping] & [Experiments, Activation, is, Activations Only] & [Subjects, Handedness, is, Right] & [Subjects, Age, is more than, 17]

The following additional database searches were conducted to help understand the composition of the results that made up the common search criteria:

[Experiments, behavioural Domain, is, Cognition, Language] & [Conditions, Stimulus, is, Auditory, All Types]

and

[Experiments, behavioural Domain, is, Cognition, Language] & [Conditions, Stimulus, is not, Auditory, All Types]

(i) *Common search results*

The common set of search criteria alone returned 997 papers with 13,949 participants and 33,153 locations. The additional searches indicate that, of these, 197 papers, 2,753 participants and 6,245 locations were in the behavioural domain of language and did not use auditory stimuli and 224 papers, 2,782 participants and 7,350 locations came from studies that used auditory stimuli. Thus, 41% of the common search locations came from studies that were about language and/or used auditory stimuli.

(*b*) *Auditory cortex*

(i) *Transverse temporal gyrus (TTG)*

In addition to the common search criteria (ESM A §2a), further search criteria for the TTG analysis described in §2b(i) was:

[Locations, TD Label, is, Gyrus, TTG]

Each "experiment" that contributed a location to the TTG was then classified. To classify the experiment as audible or not, the following meta-data was used:

[Conditions, Stimulus, is, Auditory, *]

To classify the experiment as being in the behavioural domain of language or not, the following meta-data was used:

[Experiments, behavioural Domain, is, *]

The experiments were also classified as using stimuli with meaningful semantic linguistic content or not by reading descriptions of the stimuli used in each paper.

(ii) *Passive listening*

In addition to the common search criteria (ESM A §2a), further search criteria for the passive listening analysis described in §2b(ii) were:

[Experiments, Paradigm Class, is, Passive Listening] & [Conditions, Stimulus, is, Auditory, Clicks | Noise | Pseudowords | Reversed Speech | Syllables | Tones]

and

[Experiments, Paradigm Class, is, Passive Listening] & [Conditions, Stimulus, is, Auditory, Words]

Note that [Conditions, Stimulus, is, Auditory Music] and [Conditions, Stimulus, is, Sounds (Environmental)] were excluded from the former non-word search. The number of papers returned for the this search was Auditory Clicks two, Noise five, Pseudowords three, Reversed Speech one, Syllables zero and Tones 10. See ESM A table 1 for a description of search results.

(iii) *Speech, phonology, syntax and semantics*

In addition to the common search criteria (ESM A §2a), further search criteria for the speech, phonology, syntax and semantics analysis described in §2b(iii) were:

[Experiments, behavioural Domain, is, Cognition, Language – Speech] & [Conditions, Overt Response, is not, Oral/Facial, All Types]

and

[Experiments, behavioural Domain, is, Cognition, Language – Phonology] & [Conditions, Overt Response, is not, Oral/Facial, All Types]

and

[Experiments, behavioural Domain, is, Cognition, Language – Syntax] & [Conditions, Overt Response, is not, Oral/Facial, All Types]

and

[Experiments, behavioural Domain, is, Cognition, Language – Semantics] & [Conditions, Overt Response, is not, Oral/Facial, All Types]

See ESM A table 1 for a description of search results.

(*c*) *Speech production*

In addition to the common search criteria (ESM A §2a), further search criteria for the speech production analyses described in §2c were:

[Locations, TD Label, is, Gyrus, TTG] & [Experiments, Behavioural Domain, is not, Action, Execution – Speech]

and

[Experiments, Behavioural Domain, is, Action, Execution – Speech]

and

[Experiments, Paradigm Class, is, Naming (Covert), Reading (Covert), Recitation/Repetition (Covert), Word Generation (Covert), Word Stem Completion (Covert)]

See ESM A table 1 for a description of search results.

(*d*) *Additional*

(i) *Baseline control*

In addition to the common search criteria (ESM A §2a), further search criteria for the baseline control described in §2d(i) were:

> [[Experiments, Paradigm Class, is, Passive Listening] & [Conditions, Stimulus, is, Auditory, Clicks | Noise | Pseudowords | Reversed Speech | Syllables | Tones]] & [[Experiments, behavioural Domain, is, Cognition, Language – Speech | Phonology] & [Conditions, Overt Response, is not, Oral/Facial, All Types]]

and

> [[Experiments, Paradigm Class, is, Passive Listening] & [Conditions, Stimulus, is, Auditory, Words]] & [[Experiments, behavioural Domain, is, Cognition, Language – Syntax | Semantics] & [Conditions, Overt Response, is not, Oral/Facial, All Types]]

See ESM A table 1 for a description of search results.

(ii) *Deactivations*

The common search criteria were changed. Thus, the full analysis described in §2d(ii) used the following common search criteria:

> [Experiments, Context, is, Normal Mapping] & [Experiments, Activation, is, Deactivations Only] & [Subjects, Handedness, is, Right] & [Subjects, Age, is more than, 17]

With:

> Less meaningful deactivations = [[Conditions, Stimulus, is, Auditory, All Types] & [Experiments, behavioural Domain, is, Cognition, Language – Speech | Phonology] & [Conditions, Overt Response, is not, Oral/Facial, All Types]] & [[Conditions, Stimulus, is, Auditory, Clicks | Noise | Pseudowords | Reversed Speech | Syllables | Tones] & [Conditions, Overt Response, is not, Oral/Facial, All Types]]

and

> More meaningful deactivations = [[Conditions, Stimulus, is, Auditory, All Types] & [Experiments, behavioural Domain, is, Cognition, Language – Syntax | Semantics] & [Conditions, Overt Response, is not, Oral/Facial, All Types]] & [[Conditions, Stimulus, is, Auditory, Words] & [Conditions, Overt Response, is not, Oral/Facial, All Types]]

See ESM A table 1 for a description of search results.

Table 1: BrainMap search results.

Meta-analyses	Manuscript section	BrainMap search results				
		Papers	Participants	Experiments	Conditions	Locations
**Auditory cortex**						
Transverse temporal gyrus (TTG)	§2b(i)	107	1,367	164	314	2,554
Passive listening to non-words	§2b(ii)	19	194	50	68	285
Passive listening to words	§2b(ii)	19	212	47	63	328
Speech no oral/facial	§2b(iii)	65	858	196	225	1,889
Phonology no oral/facial	§2b(iii)	29	363	79	102	575
Syntax no oral/facial	§2b(iii)	19	284	53	74	213
Semantics no oral/facial	§2b(iii)	138	1,996	423	483	3,540
**Speech production**						
TTG with no speech production	§2c	95	1,195	141	275	1,934
Speech production	§2c	48	599	102	168	1,526
Covert speech production	§2c	85	1,201	346	307	3,248
**Additional**						
Passive listening to non-words, speech and phonology no oral/facial	§2d(i)	52	665	109	191	902
Passive listening to words, syntax and semantics no oral/facial	§2d(i)	56	729	111	168	1028
Less meaningful deactivations	§2d(ii)	19	208	36	64	216
More meaningful deactivation	§2d(ii)	20	227	43	78	229

3. Auditory cortex survey

(a) Introduction

A survey was done to test the hypothesis that the proposed decrease in AC activity for meaningful linguistic stimuli and tasks compared to less meaningful stimuli and tasks is counterintuitive, even to people with cognitive neuroscience experience.

(b) Methods

Message boards were solicited for individuals with cognitive neuroscience experience. Respondents did a survey constructed to appear to be about cross-modality effects. They were asked to estimate the degree to which AC or visual cortex would be activated by auditory or visual stimuli in a typical block design fMRI or PET experiment with unimpaired participants with normal vision and hearing. Henceforth, only the two AC with auditory stimuli trials are discussed. AC was defined as A1, Heschl's gyrus, or the transverse temporal gyrus and nearby regions. In one estimation trial, auditory Condition 1 stimuli were "Spoken words (nouns and verbs, e.g., "running")" and Condition 2 stimuli were "Spoken pseudo-words ("ninurgn")." In the other estimation trial, Condition 1 stimuli were "Spoken sentences" and Condition 2 stimuli were "Spoken sentences played backwards (reversed speech)." Respondents were told that there was no visual information. They made estimations as to which of the two conditions produced more activity on a continuous sliding scale and were told to do so quickly, based on their "gut" feelings. The slider varied from "More strongly active for Condition 2" (-100) to "No difference between Conditions 1 and 2" (0) to "More strongly active for Condition 1" (+100). Respondents were told to assume that neither condition produced deactivation and that both conditions were matched for stimulus properties like duration and complexity and that participants attended to both equally. Following estimations, respondents answered five multiple choice questions pertaining to their neuroscience experience. A one sample t-test of the average of the two sets of auditory stimuli was compared with the alternative hypothesis that there was no difference between conditions.

(c) Results

Four respondents were excluded because they reported having no cognitive neuroscience experience. The status of the remaining respondents (N=100) was "other" (N=12), "undergraduate student" (N=20), "graduate student" (N=34), "postdoctoral fellow" (N=25) and "professor" (N=9). On average, this group had taken 4.67 and taught 0.90 (cognitive) neuroscience classes, read approximately 76.10 peer-reviewed journal articles containing fMRI or PET experiments and conducted or helped conduct 3.68 fMRI or PET experiments. Of respondents, 68.86% believed that meaningful auditory stimuli would engage AC more than less meaningful auditory stimuli. The participants that comprised this 68.86% believed that meaningful auditory stimuli would engage AC 34.40% more than less meaningful auditory stimuli. Across *all* respondents it was believed that meaningful auditory stimuli would engage AC 12.24% more than less meaningful auditory stimuli. This was statistically significantly different from the alternative hypothesis that there is no difference be-

tween meaningful and less meaningful stimuli (t = 2.78, df = 99, p-value = 0.007). Results do not change when participants reporting no cognitive neuroscience experience are included. In a follow-up analysis, data were divided to examine the responses of those who had conducted or helped conduct more than the mean number of fMRI or PET experiments (i.e., ≥ 4) to determine how this experience might mediate responses. More experienced respondents believed that meaningful auditory stimuli should engage AC 18.96% more than no difference between meaningful and less meaningful stimuli (t = 2.83, df = 27, p-value = 0.009).

(*d*) *Discussion*

The hypothesis that the proposed decrease in AC activity for meaningful linguistic stimuli and tasks is counterintuitive was confirmed. This is because the beliefs of people with cognitive neuroscience experience about AC functioning are inconsistent with the actual data. They generally believed that AC activity should increase for meaningful auditory stimuli whereas actual AC activity patterns decrease as determined by the meta-analyses presented in §2b.

References

[1] Cox, R. W. 1996 AFNI: software for analysis and visualization of functional magnetic resonance neuroimages. *Computers and Biomedical Research*, **29**(3), 162–173.

[2] Destrieux, C., Fischl, B., Dale, A. & Halgren, E. 2010 Automatic parcellation of human cortical gyri and sulci using standard anatomical nomenclature. *Neuroimage*, **53**(1), 1–15.

[3] Fischl, B. 2012 FreeSurfer. *Neuroimage*, **62**(2), 774–781.

[4] Holmes, C. J., Hoge, R., Collins, L., Woods, R., Toga, A. W. & Evans, A. C. 1998 Enhancement of MR images using registration for signal averaging. *Journal of computer assisted tomography*, **22**(2), 324–333. PMID: 9530404.

[5] Saad, Z. S., Reynolds, R. C., Argall, B., Japee, S. & Cox, R. W. 2004 SUMA: an interface for surface-based intra-and inter-subject analysis with AFNI. In *Biomedical imaging: Nano to macro, 2004. IEEE international symposium on*, p. 1510–1513.
